# TMEM45A enhances palbociclib resistance and cellular glycolysis by activating AKT/mTOR signaling pathway in HR+ breast cancer

**DOI:** 10.1038/s41420-025-02336-9

**Published:** 2025-02-05

**Authors:** Cui Chen, Zehong Chen, Jinze Zhao, Xinyun Wen, Hanming Yao, Zijin Weng, Huiping Xiong, Zongheng Zheng, Juekun Wu

**Affiliations:** 1 Department of Thyroid and Breast Surgery, The Third Affiliated Hospital of Sun Yat-Sen University, Sun Yat-sen University, Guangzhou, China; 2 Department of Gastrointestinal Surgery, The Third Affiliated Hospital of Sun Yat-sen University, Sun Yat-sen University, Guangzhou, China; 3 Department of Gastroenterology, Guangdong Provincial Geriatrics Institute, Guangdong Provincial People’s Hospital, Guangdong Academy of Medical Sciences, Southern Medical University, Guangzhou, China; 4 Department of Pathology, The Third Affiliated Hospital of Sun Yat-sen University, Sun Yat-sen University, Guangzhou, China

**Keywords:** Breast cancer, Cancer metabolism, DNA and RNA

## Abstract

Palbociclib, a CDK4/6 inhibitor, plays a crucial role in the treatment of HR+ breast cancer. However, resistance to palbociclib is a significant concern that merits further investigation. Our investigation identifies TMEM45A as a potential driver of palbociclib resistance and its association with increased cellular glycolysis. We demonstrate that TMEM45A is highly expressed in palbociclib-resistant breast cancer (BRCA) cells, correlating with enhanced tumor progression. Silencing TMEM45A enhances sensitivity to palbociclib, promotes cell cycle arrest and apoptosis, and inhibits the proliferation of BRCA cells. Moreover, attenuation of TMEM45A expression reduces cancer aggressiveness by decreasing the expression of EMT and glycolysis-related proteins. Subsequent gene set enrichment analysis (GSEA) confirms that TMEM45A activates the AKT/mTOR signaling pathway, which is integral to cell cycle progression and glycolysis. In a cell line-derived xenograft (CDX) mouse model, TMEM45A knockdown significantly restores sensitivity to palbociclib and suppresses tumor growth. Additionally, the use of engineered exosomes loaded with siRNA targeting TMEM45A presents a promising strategy for enhancing CDK4/6 inhibitor sensitivity without observable toxic side effects in a patient-derived xenograft (PDX) model. Collectively, our findings suggest that TMEM45A may be a therapeutic target for overcoming palbociclib resistance, and exosomal siRNA delivery could be a viable strategy for precision medicine in HR+ breast cancer.

## Introduction

Breast cancer (BRCA) is the most common malignant tumor among women, with hormone receptor-positive (HR+) BRCA being the most prevalent subtype, accounting for approximately 70% of all breast cancers [1]. The dysregulation of the cell cycle in this type of breast cancer leads to excessive cell proliferation. The application of CDK4/6 inhibitors (CDK4/6i) including palbociclib, ribociclib, and abemaciclib can improve the progression-free survival (PFS), overall survival (OS), extend chemotherapy duration, and enhance the quality of life for patients with advanced HR + BRCA [2–5]. However, despite significant improvements in the treatment of advanced disease, a substantial proportion of patients eventually develop resistance to CDK4/6i [6, 7]. Due to the emergence of treatment resistance, finding new therapeutic targets for advanced HR + BRCA is crucial for patients with CDK4/6i resistance.

The main mechanisms of resistance to CDK4/6i include abnormal activation of upstream oncogenic signaling pathways and changes in key cell cycle regulatory factors [8]. The PI3K/AKT/mTOR signaling pathway is a key upstream signaling pathway for cell cycle progression, and its abnormal activation can affect tumor progression, metabolism, invasiveness, and drug responsiveness [9]. Abnormal activation of the PI3K/AKT/mTOR signaling pathway has been proven to be a mechanism of early adaptive resistance to CDK4/6i. Alpha-specific PI3K inhibitor (PI3Ki) alpelisib has recently been approved for the treatment of PIK3CA-mutated ER+ advanced breast cancer that has progressed following endocrine therapy. PI3K inhibitors can block the early adaptation of HR+ breast cancer to CDK4/6i and prevent the acquisition of CDK4/6i resistance [10, 11]. The AKT/mTOR signaling pathway can promote glycolysis and lactate production, playing a key role in the metabolic reprogramming of cancer cells [12]. Abnormal metabolism is considered a hallmark of cancer cells; while normal cells obtain most of their energy through mitochondrial oxidative phosphorylation, cancer cells rely on aerobic glycolysis as the primary means of energy acquisition, a process in cancer cells known as the “Warburg effect” [13]. Studies have shown that by regulating the AKT/mTOR signaling pathway, the expression levels of key enzymes in the glycolysis pathway can be inhibited, thereby reducing the energy supply of tumor cells, leading to the inhibition of tumor cell proliferation and even the death of tumor cells [14].

The transmembrane (TMEM) protein family is a group of relatively unknown proteins that include one or more transmembrane domains spanning the biological membrane. TMEM45A (also known as DERP7, DNAPTP4, or FLJ10134) is a transmembrane protein composed of 275 amino acids. Studies have shown that TMEM45A is upregulated under hypoxic conditions and is closely related to chemotherapy resistance in liver, breast, head and neck, and kidney cancer cells [15, 16]. TMEM45A affects the proliferation and invasion of human ovarian cancer cells and human glioma cells [17, 18]. Studies have indicated that high expression of TMEM45A is associated with poor prognosis in patients with breast, bladder, and ovarian cancer [16, 19, 20]. However, it is not yet clear whether the expression of TMEM45A is related to the response to CDK4/6i treatment in HR + BRCA and whether TMEM45A regulates cancer metabolism.

Resistance to CDK4/6i treatment is closely related to abnormal gene regulation, and these molecules can serve as therapeutic targets. Delivering small interfering RNA (siRNA) or non-coding RNA may be a promising method for targeted therapy of resistance. Studies have shown that exosomes derived from primary cells of cancer patients can serve as an effective method for siRNA targeting delivery and enhance the sensitivity of chemotherapy and immunotherapy drugs [21, 22]. Therefore, using exosomes derived from patient tumor primary cells as siRNA carriers to deliver therapeutic siRNA to the tumor site is an effective method.

This study aims to explore the relationship between TMEM45A and the response to CDK4/6i treatment and glycolysis in HR + BRCA. We found that TMEM45A is highly expressed in cells resistant to CDK4/6i, promoting the invasive ability of breast cancer cells. Through gene set enrichment analysis (GSEA), we discovered that TMEM45A promotes cancer cells resistance to CDK4/6i and glycolysis by activating the AKT/mTOR signaling pathway. We used exosomes derived from primary BRCA tumor cells for targeted delivery of siTMEM45A in patient-derived xenograft (PDX) mouse models to enhance sensitivity to CDK4/6i. Our research results suggest that TMEM45A may be a potential therapeutic target for resistance to CDK4/6i.

## Results

### TMEM45A is associated with palbociclib resistance and glycolysis in breast cancer

**Fig. 1:**
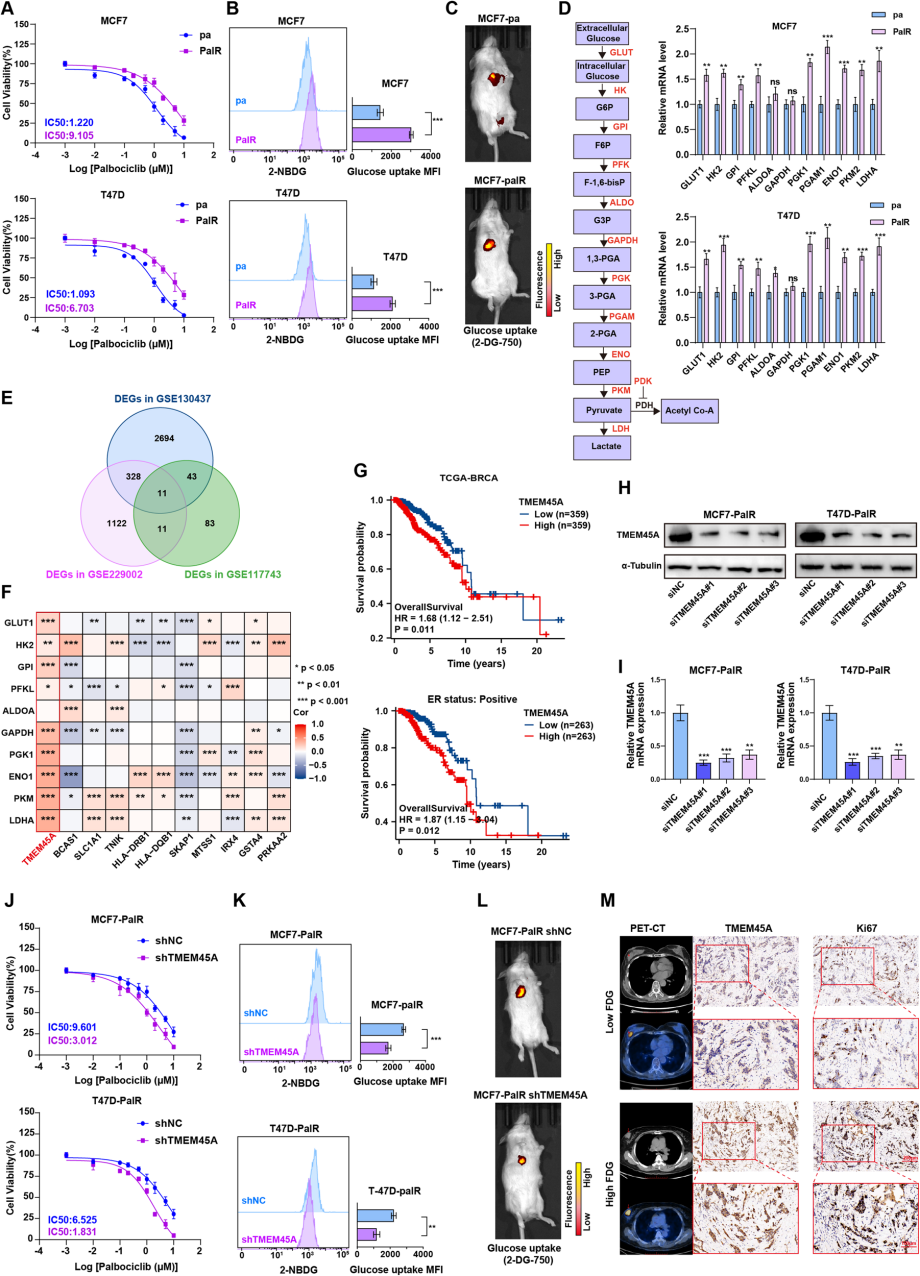
TMEM45A promotes glycolysis and palbociclib resistance in breast cancer. **A** Dose response curves of MCF7-PalR cells and T47D-PalR cells and their parental cells under palbociclib treatment for 5 days. **B** Glucose uptake activity of MCF7-PalR cells and T47D-PalR cells and their parental cells detected by the 2-NBDG probe. **C** Glucose uptake activity of MCF7-PalR cell- and their parental cell-derived xenografts tumors detected by 2-DG-750 probe in vivo. **D** RT-qPCR analysis of glycolytic gene expression in MCF7-PalR cells and T47D-PalR cells and their parental cells. *α*-Tubulin was used as a loading control for analyzing glycolysis gene expression. A schematic diagram of the aerobic glycolysis pathway is indicated on the left. **E** The upregulated differentially expressed genes (DEGs) were overlapped with the GEO datasets (GSE130437, GSE229002, GSE117743), and 11 genes may be related to palbociclib resistance. **F** Heatmap analysis based on TCGA data demonstrates a positive correlation between TMEM45A expression and glycolysis related genes. **G** Kaplan-Meier survival analysis of the overall survival of BRCA patients with high or low expression levels of TMEM45A in TCGA. **H**, **I** Western blotting (**H**) and RT-qPCR (**I**) indicated effective TMEM45A knockdown. **J** Dose response curves of MCF7-PalR and T47D-PalR cells with or without TMEM45A knockdown under palbociclib treatment for 5 days. **K** Glucose uptake activity of MCF7-PalR and T47D-PalR cells detected by the 2-NBDG probe with or without knockdown of TMEM45A. **L** Detection of glucose uptake in MCF7-PalR cell-derived xenograft tumor by the 2-DG-750 probe in vivo with or without knockdown of TMEM45A. **M** Correlation of TMEM45A expression with 18F-FDG accumulation and the expression levels of Ki67 in BRCA patients. *P < 0.05, **P < 0.01, ***P < 0.001. ns, not significance.

To identify potential mechanisms of resistance to CDK4/6i, we obtained drug-resistant cell lines MCF7-PalR and T47D-PalR by culturing MCF7 and T47D in gradually increasing concentrations of palbociclib-containing medium (starting from 0.01 μM up to 5 μM) for 6 months. The dose-response analysis showed that compared with the parental cells, MCF7-PalR and T47D-PalR exhibited more than 6-fold increased resistance to palbociclib (Fig. 1A). Aerobic glycolysis represents a vital feature of the energy metabolism in cancer cells. During the process of palbociclib resistance, the metabolic characteristics of tumor cells may have changed. We investigated the impact of glycolysis on palbociclib resistance by measuring the glucose uptake of the cells, and the results indicated that the glucose uptake of MCF7-PalR and T47D-PalR was increased compared with the parental cells (Fig. 1B). Then we used cell line-derived xenograft (CDX) model to monitor glucose uptake in vivo. Parental MCF7 and drug-resistant MCF7-PalR cells were implanted into the mammary fat pads of NOD/SCID female mice and 2-DeoxyGlucosone (DG)-750 probe was injected to visualize the tumor glucose uptake. The MCF7-PalR tumor had more glucose uptake compared to the parental tumor (Fig. 1C). Similarly, RT-qPCR analysis demonstrated that several glycolysis-related genes (GLUT1, HK2, GPI, PFKL, ALDOA, GAPDH, PGK1, PGAM1, ENO1, PKM2, LDHA) were markedly upregulated in palbociclib-resistant BRCA cells (Fig. 1D). Subsequently, we identified three datasets related to palbociclib resistance from the GEO database (GSE130437, GSE229002, GSE117743), and by intersecting the upregulated DEGs from the three datasets, we found that 11 genes (TMEM45A, BCAS1, SLC1A1, TNIK, HLA-DRB1, HLA-DQB1, SKAP1, MTSS1, IRX4, GSTA4, PRKAA2) were abnormally upregulated and may be related to palbociclib resistance (Fig. 1E). After conducting a correlation analysis of the 11 candidate genes with glycolysis-related genes, TMEM45A was found to be a key gene that may be related to both palbociclib resistance and glycolysis (Fig. 1F). Therefore, we further investigated the role of TMEM45A in glycolysis and palbociclib resistance. Pan-cancer analysis of TCGA data has shown that TMEM45A is highly expressed in several cancers, including breast cancer. High expression of TMEM45A is linked to poorer overall survival (OS) in breast cancer, particularly in ER+ breast cancer. Furthermore, high TMEM45A expression is connected to distant metastasis in breast cancer (Figs. 1G and S1A-D).

To investigate the role of TMEM45A in breast cancer, we established BRCA cell lines with TMEM45A knockdown using siRNA targeting TMEM45A (siTMEM45A) and a negative control siRNA (siNC). The efficiency of knockdown was confirmed by both western blot and RT-qPCR analyses (Figs. 1H–I and S2A, B). Based on the knockdown efficiency, we selected the siTMEM45A#1 sequence to construct stable cell lines, which were named shTMEM45A and shNC for subsequent experiments. Dose-response experiments revealed that the IC50 of palbociclib was decreased in TMEM45A-knockdown BRCA cells (Fig. 1J). Consistently, a reduction in glucose uptake was observed in TMEM45A-knockdown BRCA cells both in vitro and in vivo, indicating a potential impact on glycolysis (Fig. 1K, L). Furthermore, immunohistochemical staining of BRCA patient tissues correlated with PET/CT findings showed that patients with high TMEM45A expression exhibited elevated maximum standardized uptake values (SUV_max_) on 18F-PET/CT scans (Fig. 1M). Notably, the expression of TMEM45A was positively correlated with the expression of Ki67, implying a possible link between TMEM45A expression and the proliferative capacity of BRCA cells (Figs. 1M and S3A, B). Taken together, these findings implied that TMEM45A contributes to glycolysis enhancement in palbociclib-resistant BRCA cells, which might account for the resistance to palbociclib.

### TMEM45A inhibits the cell cycle arrest, apoptosis and promote proliferation in BRCA cells

**Fig. 2:**
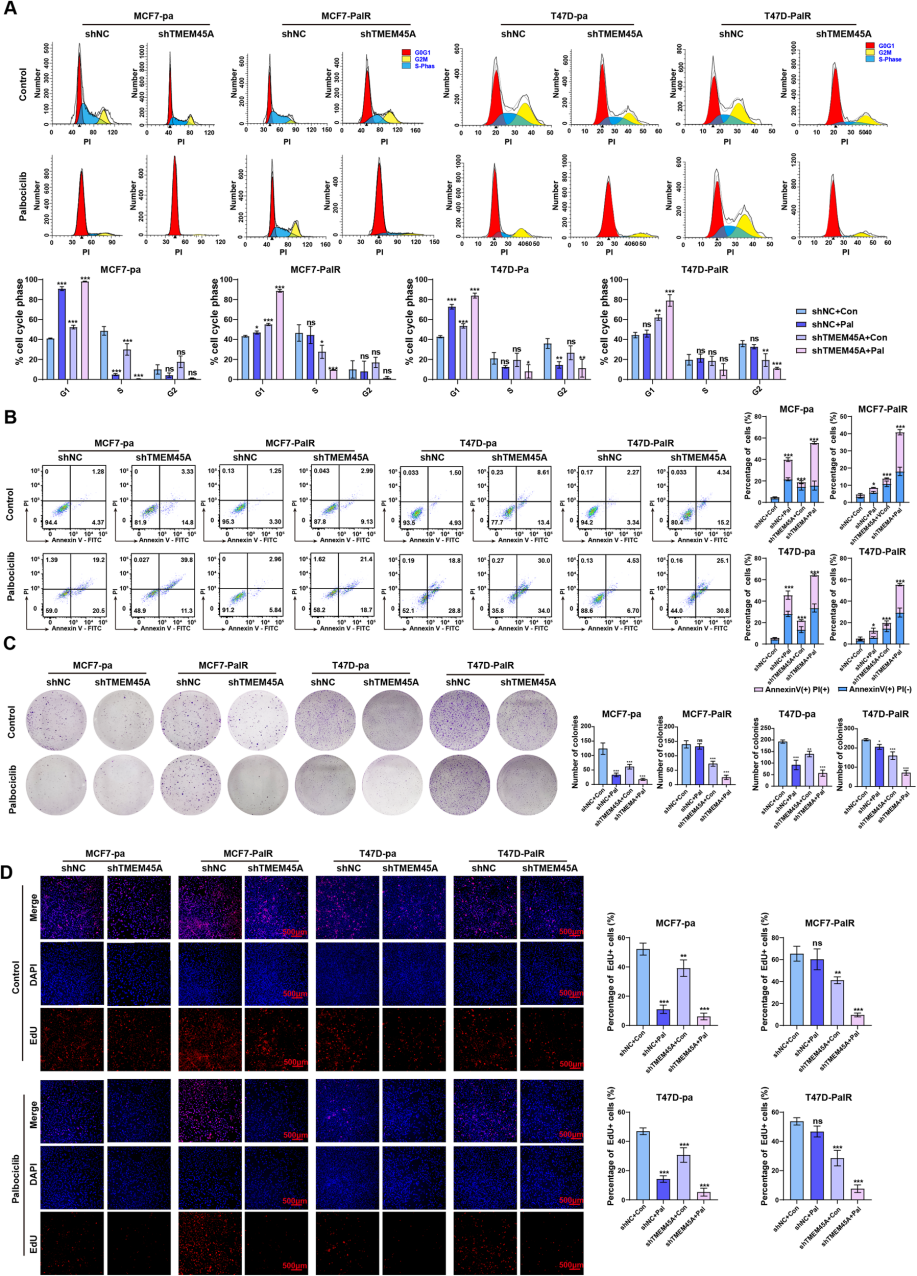
TMEM45A inhibits the cell cycle arrest, apoptosis and promotes proliferation in BRCA cells. **A** Cell cycle analysis by Flow Cytometry showing the cell cycle distribution under 24 h of treatment with vehicle (DMSO) or 1 μM palbociclib and statistical analysis are shown below. **B** Cell apoptosis assay by Annexin-V/PI dual staining under 24 h of treatment with vehicle (DMSO) or 1 μM palbociclib and statistical analysis are shown on the right side. **C** The results of the colony formation assay under 14 days treatment with vehicle (DMSO) or 1 μM palbociclib and statistical analysis are shown on the right side. **D** 5-ethynyl-2′-deoxyuridine (EdU) incorporation assay show the proliferative ability of BRCA cells under 24 h of treatment with vehicle (DMSO) or 1 μM palbociclib and statistical analysis are shown on the right side. *P < 0.05, **P < 0.01, ***P < 0.001. ns, not significance.

To assess the impact of TMEM45A on the cell cycle, flow cytometry was utilized to analyze changes in the cell cycle following TMEM45A knockdown. Downregulation of TMEM45A mRNA induce a higher proportion of cells to arrest in the G1 phase, while the suppression of TMEM45A combined with palbociclib treatment significantly increased G1 phase arrest in vitro, even in palbociclib-resistant cell lines (Fig. 2A). To investigate the effect of TMEM45A on apoptosis, we utilized flow cytometry to assess the changes in Annexin V+ cell populations. Knockdown of TMEM45A increased the percentage of Annexin V+ cells, and the combination of TMEM45A knockdown with palbociclib treatment significantly increased the proportion of Annexin V+ cells in both parental and palbociclib-resistant BRCA cells (Fig. 2B). The influence of TMEM45A on cell proliferation was assessed using colony formation assays and EdU incorporation assays. TMEM45A knockdown significantly impaired the ability of cells to form colonies and diminished EdU incorporation. Furthermore, the reduction of TMEM45A expression in conjunction with palbociclib treatment synergistically decreased the colony-forming capacity and reduced EdU incorporation of parental and palbociclib-resistant BRCA cells (Fig. 2C, D). Collectively, these findings suggest that TMEM45A knockdown enhanced the effects of palbociclib treatment on cell cycle arrest, apoptosis induction, and proliferation inhibition across both parental and resistant BRCA cells.

### TMEM45A facilitates Epithelial-Mesenchymal Transition (EMT) and enhances glycolysis in BRCA cells

**Fig. 3:**
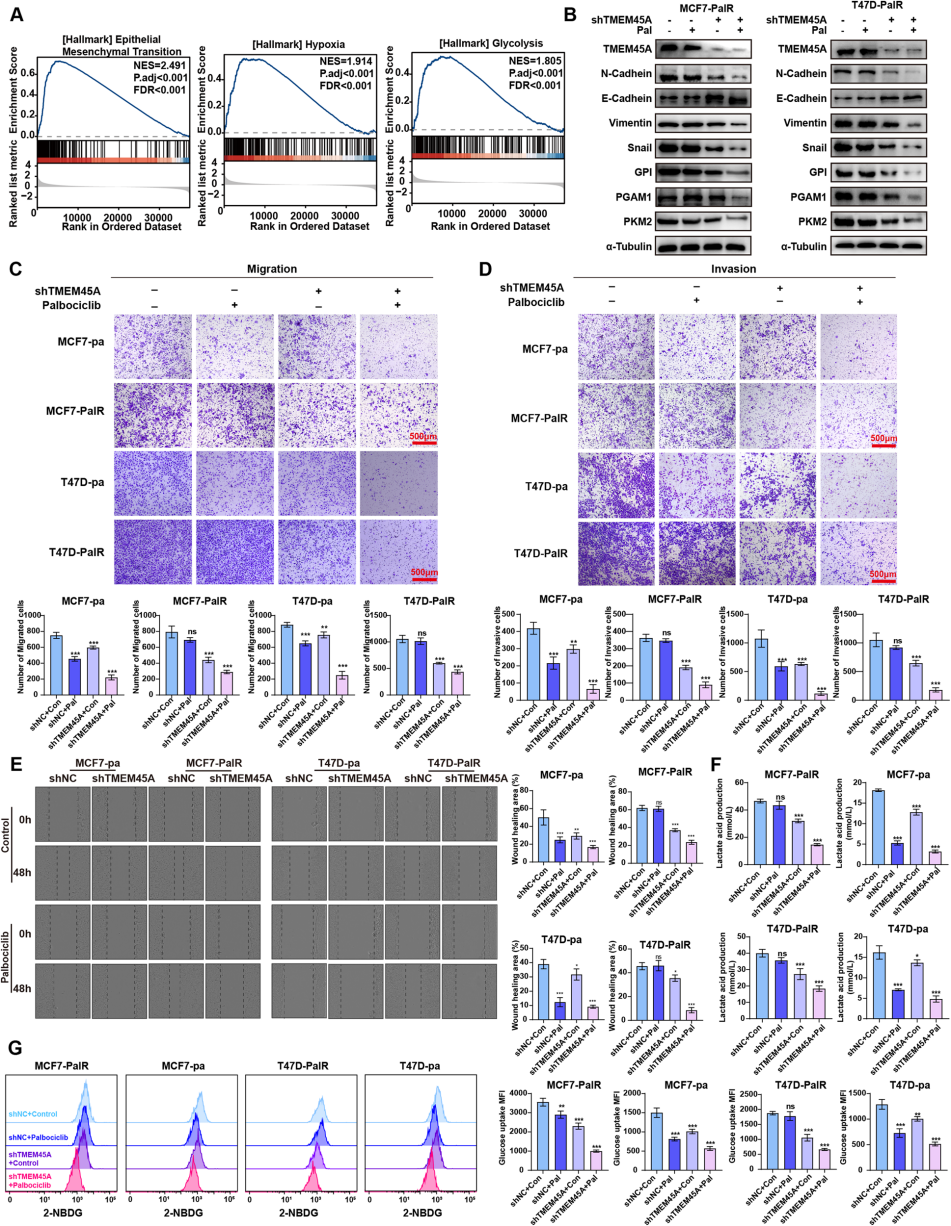
TMEM45A promote EMT and glycolysis in BRCA cells. **A** GSEA enrichment plot derived from the TCGA dataset shows significant enrichment of Epithelial Mesenchymal Transition (EMT), Hypoxia, and Glycolysis in the high expression group of TMEM45A of BRCA. **B** Western blot showing the change of EMT and Glycolysis related proteins in MCF7-PalR cells and T47D-PalR cells with or without 1 μM palbociclib treatments and TMEM45A knockdown. **C**, **D** Migration (**C**) and invasion (**D**) assays of BRCA cells with or without 1 μM palbociclib treatments and TMEM45A knockdown. Statistical analyses are shown below. **E** Wound healing assays of BRCA cells with or without 1 μM palbociclib treatments and TMEM45A knockdown. Statistical analyses are shown on the right side. **F**, **G** Lactate acid production (**F**) and glucose uptake abilities (**G**) of BRCA cells with or without 1 μM palbociclib treatments and TMEM45A knockdown. *P < 0.05, **P < 0.01, ***P < 0.001. ns, not significance.

Gene set enrichment analysis (GSEA) revealed that elevated TMEM45A expression correlates with the upregulation of pathways involved in Epithelial-Mesenchymal Transition (EMT), Hypoxia, and Glycolysis (Fig. 3A). Western blot analysis demonstrated that treatment with palbociclib alone did not result in a significant change in the expression of proteins associated with EMT and glycolysis in MCF7-PalR and T47D-PalR cells. However, the knockdown of TMEM45A led to a reduction in EMT markers including N-cadherin, Vimentin, and Snail, while concurrently increasing the expression of E-cadherin. Additionally, the knockdown of TMEM45A was associated with decreased levels of glycolysis-related genes such as GPI, PGAM1, and PKM2. Notably, the subsequent administration of palbociclib following TMEM45A knockdown further downregulated the expression of proteins related to EMT and glycolysis (Fig. 3B). These findings suggest that TMEM45A plays a pivotal role in the regulation of EMT and glycolysis.

Additionally, we utilized the transwell assay to evaluate the migratory and invasive capabilities of tumor cells. Treatment with palbociclib resulted in the suppression of migration and invasion in the parental MCF7 and T47D cells, while it had no significant effect on the resistant strains. Knockdown of TMEM45A was found to inhibit the migratory and invasive abilities of both the parental and palbociclib-resistant MCF7 and T47D cells, while the combination of TMEM45A knockdown and palbociclib treatment led to significant inhibition of these cells’ migration and invasion abilities (Fig. 3C, D). Consistently, wound healing assay also demonstrated that TMEM45A knockdown inhibits the migration ability of BRCA cells (Fig. 3E). Subsequently, we assessed the influence of TMEM45A knockdown on cellular glycolysis activity by quantifying glucose uptake and lactate production. Compared to the palbociclib treatment, the combination of TMEM45A knockdown and palbociclib significantly reduced the lactate production and glucose uptake in BRCA cells, as shown in Fig. 3F, G. Taken together, these results suggest that TMEM45A plays a role in promoting EMT and increasing glycolysis in BRCA cells, thereby contributing to their ascending malignant biological behaviors.

### TMEM45A promotes palbociclib resistance and glycolysis in breast cancer by activating the AKT/mTOR signaling pathway

**Fig. 4:**
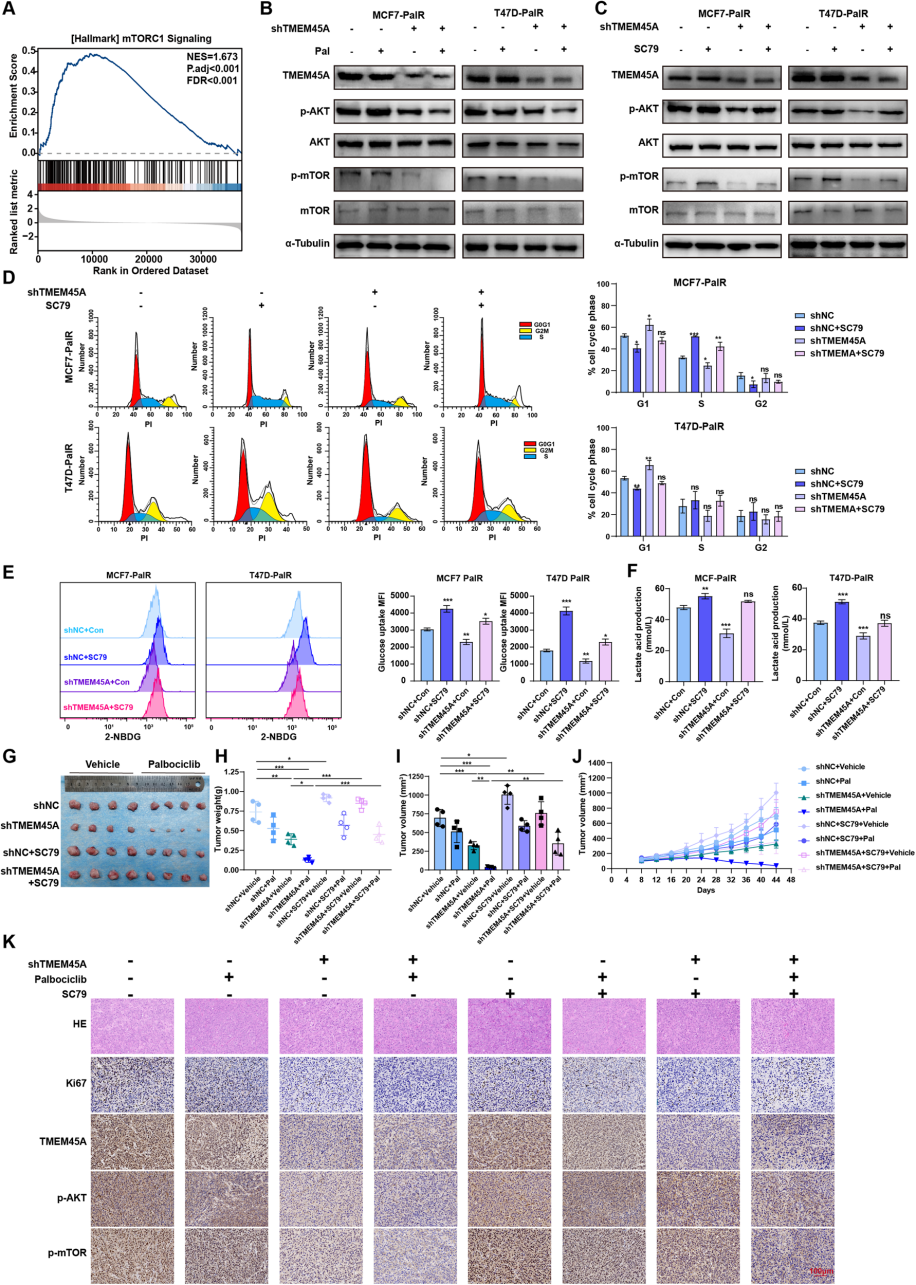
TMEM45A enhances palbociclib resistance and glycolysis in BRCA cells through activation of the AKT/mTOR signaling pathway. **A** GSEA enrichment plot derived from the TCGA dataset shows significant enrichment of mTOR1 in BRCA with high TMEM45A expression. **B** Western blot shows AKT/mTOR pathway expression in MCF7-PalR cells and T47D-PalR cells with or without 1 μM palbociclib treatment and TMEM45A knockdown. **C** Western blot shows AKT/mTOR pathway expression in MCF7-PalR cells and T47D-PalR cells with or without 10 μM SC79 treatment and TMEM45A knockdown. **D** Cell cycle analysis of MCF7-PalR and T47D-PalR cells with or without 10 μM SC79 treatment and TMEM45A knockdown. Statistical analyses are shown on the right side. **E** Glucose uptake of MCF7-PalR and T47D-PalR cells treated with or without 10 μM SC79 treatment and TMEM45A knockdown. Statistical analyses are shown on the right side. **F** Lactate acid production in MCF7-PalR and T47D-PalR cells treated with or without 10 μM SC79 treatment and TMEM45A knockdown. **G** Representative images of the tumors of the CDX mice models. **H** Statistical analysis of tumor weight in different groups. **I**, **J** Statistical analysis (**I**) and growth curve (**J**) of tumor volume in different groups. **K** Representative images of IHC staining for HE, Ki67, TMEM45A, p-AKT and p-mTOR in CDX tumor. *P < 0.05, **P < 0.01, ***P < 0.001. ns, not significance.

To further explore the mechanism of TMEM45A in BRCA, we performed a gene set enrichment analysis (GSEA) and found that high expression of TMEM45A may be associated with the upregulation of the mTORC1 signaling pathway (Fig. 4A). Western blotting results showed that compared to the parental cells, the MCF7-PalR and T47D-PalR exhibited activation of the AKT/mTOR signaling pathway, with increased expression levels of phosphorylated AKT (p-AKT) and phosphorylated mTOR (p-mTOR) (Figure S4). In MCF7-PalR and T47D-PalR cells, treatment with palbociclib had no effect on the phosphorylation levels of the AKT/mTOR pathway. However, knockdown of TMEM45A reduced the expression levels of p-AKT and p-mTOR and restored the inhibitory effect of palbociclib on AKT/mTOR signaling pathway in the resistant cells (Fig. 4B). The inhibitory effect on the AKT/mTOR signaling pathway after TMEM45A knockdown was reversed using the pathway activator SC79 (Fig. 4C). Flow cytometry analysis revealed that treatment with SC79 reduced the G1 phase cell cycle arrest induced by TMEM45A knockdown (Fig. 4D) and restored the glucose uptake levels and lactate production in BRCA cells (Fig. 4E, F), demonstrating that TMEM45A contributes to drug resistance and promotes cellular glycolysis through the AKT/mTOR pathway.

To validate our findings in vivo, subcutaneous xenograft tumors were established by implanting MCF7-palR-shNC and MCF7-palR-shTMEM45A cells into the mammary fat pads of NOD/SCID mice. When the tumor volume in the mice reached approximately 100 mm^3, the mice bearing MCF7-palR-shNC and MCF7-palR-shTMEM45A cells were randomly divided into four groups, which were then treated with a vehicle, palbociclib alone, SC79 alone, and a combination of palbociclib and SC79 for a period of 5 weeks. TMEM45A knockdown combination with palbociclib treatment significantly inhibited tumor growth in vivo, suggesting a synergistic effect in the inhibition of tumor progression. Nonetheless, SC79 treatment has been shown to counteract the suppressive effects induced by the TMEM45A knockdown and palbociclib therapy, demonstrating that TMEM45A confers resistance to palbociclib by activating the AKT/mTOR signaling pathway (Fig. 4G–J). The body weight of mice treated with different regimens remained stable (Fig. S5). Furthermore, we verified our findings through immunohistochemical sections of tumor tissue, which indicated that TMEM45A knockdown could suppress the levels of p-AKT and p-mTOR, and the use of SC79 could attenuate the inhibitory effect of TMEM45A knockdown on the pathway (Fig. 4K). In summary, TMEM45A may mediate palbociclib resistance in BRCA cells through activation of the AKT/mTOR signaling pathway.

### Construction and characterization of the BRCA primary cell-derived engineered exosomes

**Fig. 5:**
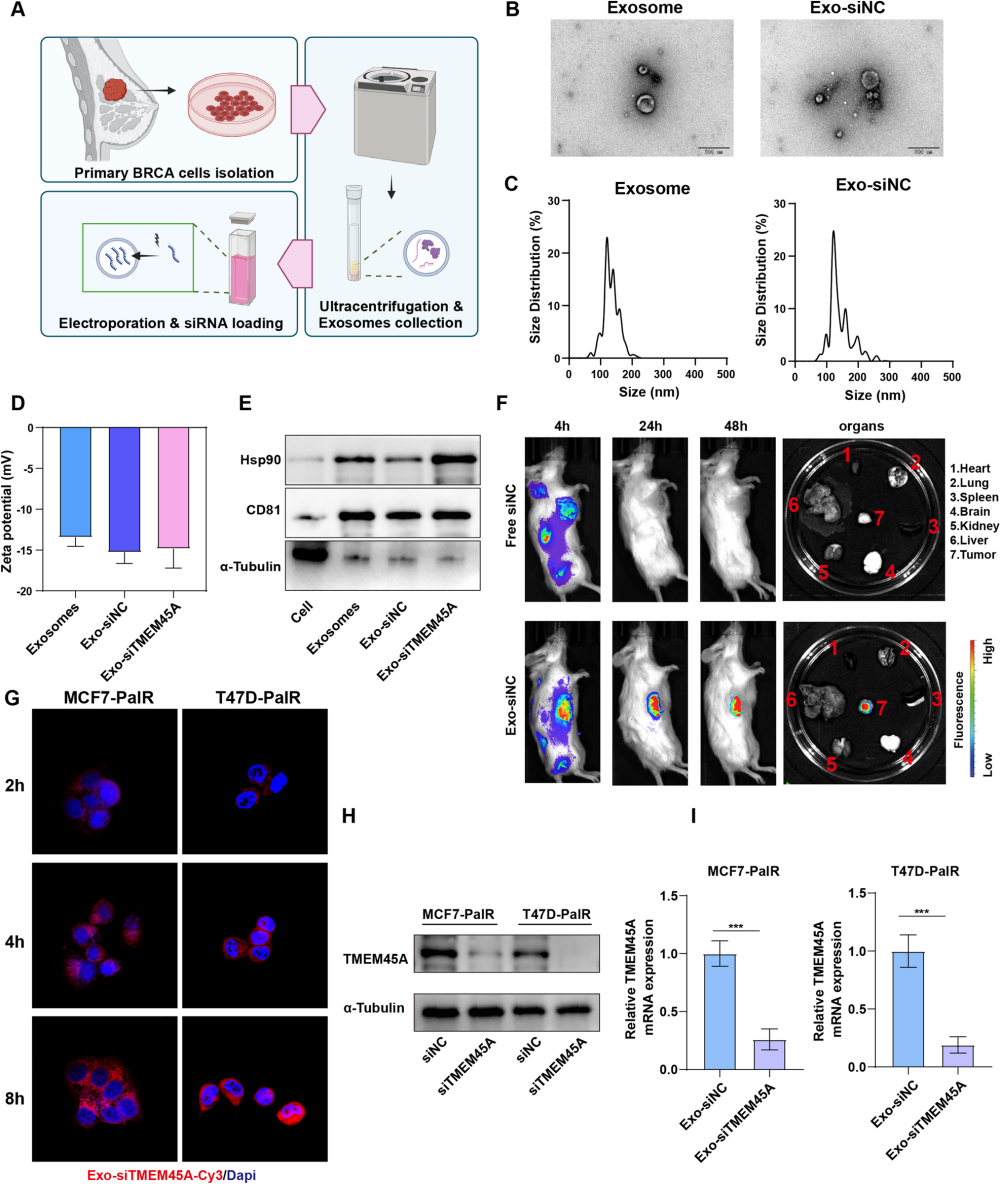
Construction and characterization of the BRCA primary cell-derived engineered exosomes. **A** Schematic diagram of primary BRCA cells culture and exosomes isolation. **B**, **C** TEM photographs (**B**) of exosomes and exosomes loaded with siNC (Exo-siNC), and their size distribution graphs (**C**). **D** The zeta potential measurements of exosomes, Exo-siNC and Exo-siTMEM45A. **E** Western blot analysis of exosome markers. **F** The distribution of biologically engineered exosomes within the PDX model. **G** Cellular internalization of engineered exosomes was evaluated using confocal microscopy. **H**–**I** Western blotting (**H**) and RT-qPCR (**I**) revealed the knockdown capacity of the engineered exosomes in vitro. ***P < 0.001.

Small interfering RNAs (siRNAs) offer a targeted approach to downregulate pathogenic genes, showing considerable promise in treating human diseases, particularly malignancies. Exosomes, as a delivery vehicle for siRNAs, exhibit high potential due to their ability to effectively transport therapeutic molecules across biological barriers. To obtain exosomes with high biocompatibility, low immunogenicity, and active targeting capabilities, we extracted primary BRCA tumor cells from tissue samples of breast cancer patients and then isolated and purified exosomes derived from primary BRCA tumor cells (Fig. 5A). Exosomes were transfected with siRNA-negative controls (siNC), and their morphology and size were characterized using electron microscopy and nanoparticle tracking analysis (Fig. 5B–D). Western blotting showed that the exosome markers Hsp90 and CD81 did not significantly differ among the control exosomes, Exo-siNC, and Exo-siTMEM45A, were scarcely present in primary tumor cells, while α-Tubulin was expressed in primary cells but at lower levels in exosomes (Fig. 5E). We then determined the loading efficiency of siRNA in exosomes using Cy3-labeled siRNA. The fluorescence intensity at various siRNA concentrations was measured using a microplate reader, and a standard curve was plotted accordingly. Our calculations revealed that on average, 10^10^ engineered exosomes contained 0.202 ± 0.013 μg of siRNA (Fig. S6A). Additionally, the polydispersity index (PDI) showed no statistical difference between groups (Fig. S6B).

To investigate the in vivo uptake and distribution of engineered exosomes, Cy3-labeled free siNC-Cy3 and Exo-siNC-Cy3 were intravenously injected into PDX-bearing mice. In vivo fluorescence imaging revealed that the engineered exosomes accumulated and persisted within the tumor, whereas free siRNA was completely degraded within 24 hours (Fig. 5F). This indicates that engineered exosomes exhibit favorable targeting and stability in vivo. Then we evaluated the cellular uptake of engineered exosomes in BRCA cells using confocal microscopy. Fluorescence was evident in the cytoplasm of recipient cells after 2, 4, and 8 hours, demonstrating the engineered exosomes could be internalized by BRCA cells (Fig. 5G). Western blotting and RT-qPCR showed the expression of TMEM45A in BRCA cells was significantly downregulated after Exo-siTMEM45A treatment, suggesting a promising knockdown efficiency of the engineered exosomes (Fig. H-I).

To evaluate the stability of engineered exosomes under in vitro storage conditions, the exosomes were resuspended in PBS and maintained at 4 °C. Over a period of 48 hours, no significant changes in exosome diameter were detected (Fig. S6C). Similarly, we simulated in vivo circulatory conditions using 10% FBS/PBS at 37 °C and observed that the diameter of engineered exosomes remained stable over 48 hours, suggesting that these exosomes can maintain stability within the bloodstream (Fig. S6D). Finally, to assess the biocompatibility of engineered exosomes in blood, we incubated varying concentrations of exosomes with peripheral blood from NOD/SCID mice and no hemolytic reactions were observed, suggesting engineered exosomes may have good biocompatibility in the bloodstream (Fig. S6E). In summary, we have developed the BRCA patient-derived engineered exosome successfully by loading siTEME45A without altering their inherent structure and physicochemical properties.

### Antitumor function of bioengineered exosomes in the PDX model

**Fig. 6:**
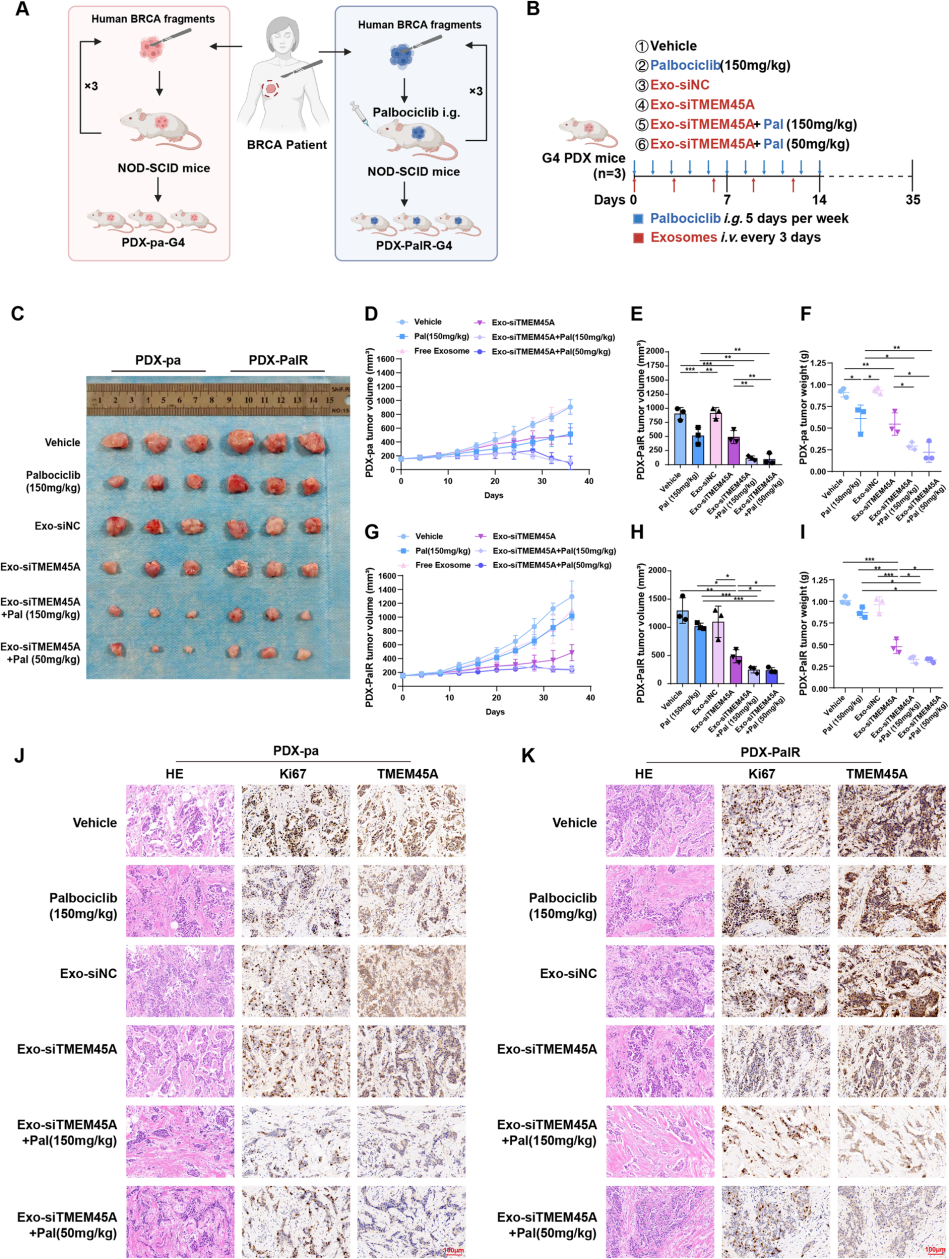
The tumor-suppressive role of engineered exosomes in PDX model. **A** Schematic diagram of patient-derived xenograft (PDX) model establishment. **B** Schematic diagram of palbociclib and engineered exosomes treatment schedule. **C** Representative images of tumors in different groups. **D**, **E** Growth curve (**D**) and statistically analyses (**E**) of the PDX-pa tumor volume. **F** Tumor weight of PDX-pa tumor. **G**, **H** Growth curve (**G**) and statistically analyses (**H**) of the PDX-PalR tumor volume. **I** Tumor weight of PDX-PalR tumor. **J**, **K** Immunohistochemical staining of HE, Ki67, TMEM45A of PDX-pa (**J**) and PDX-PalR (**K**) tumor. *P < 0.05, **P < 0.01, ***P < 0.001.

We explored the therapeutic effects of exosomes in vivo by constructing a patient-derived xenograft (PDX) model. Tumor tissue samples from an untreated ER+, PR+, HER2- breast cancer patient were collected and implanted into the subcutaneous tissue of NOD/SCID mice. Mice with palbociclib resistance were obtained through continuous exposure to palbociclib [23], as shown in Fig. 6A, and the grouping and treatment methods of the mice are depicted in Fig. 6B. In PDX-pa mice, the use of palbociclib alone (150 mg/kg) and Exo-siTMEM45A alone could inhibit tumor growth, while the combined use of Exo-siTMEM45A and palbociclib (150 mg/kg) significantly suppressed tumor size. Additionally, reducing the dosage of palbociclib used (50 mg/kg) combining Exo-siTMEM45A was also found to be effective in suppressing tumor growth. In the PDX-PalR mouse model exhibiting resistance to palbociclib, monotherapy with palbociclib (150 mg/kg) did not significantly reduce tumor size. However, the combination of Exo-siTMEM45A and palbociclib (150 mg/kg), even at low concentrations (50 mg/kg), markedly suppressed tumor growth (Fig. 6C–I). The result implied that the combination of Exo-siTMEM45A and palbociclib significantly benefited tumor CDK4/6 inhibitor therapy and the delivery of siTMEM45A by engineered exosomes might significantly reduce the use of palbociclib. IHC staining showed that Exo-siTMEM45A knocked down the level of TMEM45A in PDX tumor tissue, indicating that engineered exosomes had a good knockdown efficiency in vivo. Combined use of palbociclib and Exo-siTMEM45A could also inhibit the expression of proliferation marker Ki67 in tumor tissue (Fig. 6J, K).

To determine whether engineered exosomes have any side effects in vivo, we collected blood samples at the end of the PDX experiment for the detection of ALT, AST, BUN, and CREA. The results showed no significant differences between groups, and there were also no obvious statistical differences in the body weight of mice across the groups (Fig. S7A-D). Major organs from the mice were also collected and subjected to HE staining at the end of the experiment, as shown in Fig. S7E, suggest that engineered exosomes exhibit no significant toxicity in organs. Therefore, engineered exosomes exhibited no obvious toxic effects in the mouse model.

**Fig. 7:**
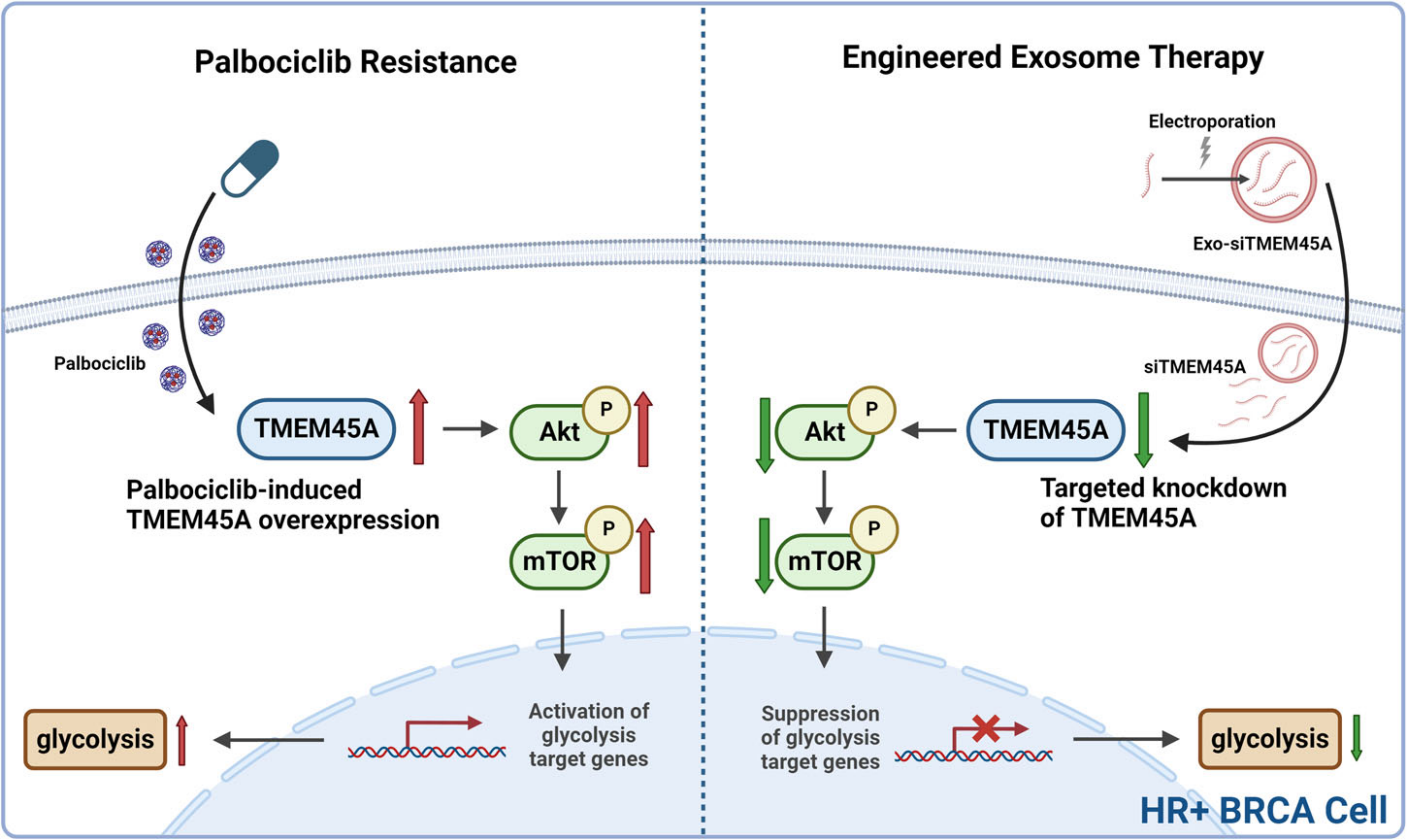
The schematic figure of the study.

In summary, we report that TMEM45A promotes glycolysis and palbociclib resistance via the AKT/mTOR signaling pathway in BRCA, leading to enhanced tumor progression. Subsequently, engineered exosomes can effectively target TMEM45A and enhance sensitivity to palbociclib in the PalR-PDX model. Our findings suggest that TMEM45A may be a potential therapeutic target for overcoming palbociclib resistance in HR+ breast cancer (Fig. 7).

## Discussion

Although CDK4/6i are widely acknowledged as pivotal therapeutic drugs for patients with advanced HR + BRCA, their therapeutic efficacy and treatment duration are significantly constrained by the development of drug resistance [24]. Consequently, it is urgent to explore the mechanisms of resistance to CDK4/6i. In this study, we established palbociclib resistant cell lines and, through both in vitro and in vivo validation, identified TMEM45A as a potential therapeutic target for overcoming palbociclib resistance in BRCA. Targeted tumor energy metabolism therapy has received widespread attention and has potential clinical significance. Tumor cells obtain energy through glycolysis in an aerobic environment, a process known as aerobic glycolysis. Targeting aerobic glycolysis is a novel approach to cancer treatment. This study focused on the biological role of TMEM45A in promoting BRCA energy metabolism and palbociclib resistance. Mechanistically, TMEM45A mediated palbociclib resistance and promoted glycolysis by activating the AKT/mTOR signaling pathway. Based on these results, we constructed exosomes from patient-derived primary cells to deliver siRNA targeting TMEM45A. The exosome delivery system increased sensitivity to palbociclib in a palbociclib-resistant PDX model, indicating that TMEM45A is a promising therapeutic target for BRCA palbociclib resistance.

TMEM45A is a protein with significant potential in the field of cancer research. Our study found that TMEM45A can inhibit the cell cycle arrest and apoptosis during tumor progression and is related to the migration and invasion capabilities of tumors, consistent with the research by GUO et al. [16, 18]. Although it has been shown that TMEM45A can promote cisplatin drug resistance under hypoxic conditions, its relationship with palbociclib resistance is unclear [25, 26]. During the process of drug resistance, changes may occur in the metabolic state of tumor cells. Aerobic glycolysis is an important characteristic of tumor cell energy metabolism. We detected glucose uptake rates and lactate production levels between resistant cells and parental cells and found that palbociclib-resistant cells are more dependent on glycolysis, consistent with the metabolomics research by Juliana et al. [27]. This study mainly explores the role of TMEM45A in palbociclib resistance and glycolysis. We performed GSEA analysis on the BRCA data in the TCGA database and found that TMEM45A may promote palbociclib resistance and glycolysis by activating the AKT/mTOR pathway. It is well known that the AKT/mTOR signaling pathway is abnormally activated in CDK4/6 inhibitor resistance and is related to early resistance to CDK4/6 inhibitor treatment [28, 29]. Protein kinase B (AKT), known as the “Warburg kinase,” promotes tumor cell metabolic reprogramming and increases cell invasiveness [12]. The AKT/mTOR signaling pathway is related to the regulation of various biological behaviors in tumor cells and plays a key role in the occurrence and development of BRCA [30]. Therefore, we speculate that TMEM45A is a mechanism of acquired resistance in breast cancer patients by activating the AKT/mTOR signaling pathway and enhancing glycolysis.

Gene therapy based on siRNA holds promise as a therapeutic strategy for breast cancer. Despite the identification of targets associated with palbociclib resistance in BRCA, the challenge of safely and effectively delivering siRNA to target cells remains a barrier to the clinical application of this approach. Exosomes are increasingly becoming significant as carriers for siRNA in biomedical research. Exosomes are nanoscale vesicles secreted by cells and possess natural biocompatibility and low immunogenicity, making them ideal candidates for gene therapy vectors [31, 32]. They are capable of long-distance transport within the body and can protect siRNA from degradation by nucleases [33]. In this study, we utilized exosomes derived from patient tumor primary cells as carriers for siRNA to deliver therapeutic siTMEM45A to the tumor site. This approach specifically reduces the expression of TMEM45A within tumor cells, minimizing the toxicity and immune response associated with external delivery vectors.

Although we propose that targeted delivery of siRNA via exosomes derived from primary tumor cells of BRCA patients can effectively suppress the expression of TMEM45A in tumor cells, this method has not yet been translated and validated in clinical settings and thus has certain limitations. Furthermore, while this study confirms that TMEM45A plays a role in restoring sensitivity to palbociclib, the role of this target in resistance to other CDK4/6 inhibitors requires further substantiation. In summary, our study has identified the TMEM45A/AKT/mTOR axis as a mediator of palbociclib resistance in breast cancer by promoting glycolysis and EMT in BRCA cells, highlighting its potential as a therapeutic target.

## Materials and methods

### Clinical data and samples

Tumor samples were obtained from 50 patients with HR + BRCA at the Third Affiliated Hospital of Sun Yat-sen University, and the tissue specimens were embedded for IHC. Among them, 32 patients had Positron Emission Tomography/Computed Tomography (PET/CT) examination results. Tumor specimens from 1 patient were collected to construct Patient-Derived Xenograft (PDX) models and to extract primary tumor cells for subsequent experiments. This study was reviewed and approved by the Ethics Committee of the Third Affiliated Hospital of Sun Yat-sen University, with the approval number: II2023-088-01. All the patients enrolled in this study agreed and signed informed consent.

### Bioinformatic Analysis

Analysis of key genes associated with resistance to palbociclib treatment in BRCA cells was conducted using the GEO datasets (GSE29002, GSE117743, and GSE130437) and GEO2R software. Genes with a log2 fold change (log2FC)＞1 and an adjusted *p*-value < 0.05 were defined as upregulated differentially expressed genes in the datasets. Pan-cancer analysis was performed using data from The Cancer Genome Atlas (TCGA) to evaluate the expression patterns of genes across various cancer types. The abbreviations and full names of the cancers mentioned are listed in Table 1. Gene Set Enrichment Analysis (GSEA) was employed to analyze the high and low expression groups of TMEM45A in breast cancer tissues from TCGA based on the “MSigDB hallmark gene sets” within the Molecular Signatures Database. A normalized enrichment score (NES) of ≥ 1.5, a normalized *p*-value less than 0.05, and a False Discovery Rate (FDR) adjusted q-value <0.25 were considered statistically significant.

**Table 1:** The abbreviations and full names.

Abbreviations	full names
HR+	hormone receptor-positive
CDK4/6i	CDK4/6 inhibitors
siRNA	small interfering RNA
PDX	patient-derived xenograft
BRCA	Breast cancer
ACC	Adrenocortical carcinoma
BLCA	Bladder Urothelial Carcinoma
CESC	Cervical squamous cell carcinoma and endocervical adenocarcinoma
CHOL	Cholangio carcinoma
COAD	Colon adenocarcinoma
DLBC	Lymphoid Neoplasm Diffuse Large B-cell Lymphoma
ESCA	Esophageal carcinoma
GBM	Glioblastoma multiforme
HNSC	Head and Neck squamous cell carcinoma
KICH	Kidney Chromophobe
KIRC	Kidney renal clear cell carcinoma
KIRP	Kidney renal papillary cell carcinoma
LAML	Acute Myeloid Leukemia
LGG	Brain Lower Grade Glioma
LIHC	Liver hepatocellular carcinoma
LUAD	Lung adenocarcinoma
LUSC	Lung squamous cell carcinoma
MESO	Mesothelioma
OV	Ovarian serous cystadenocarcinoma
PAAD	Pancreatic adenocarcinoma
PCPG	Pheochromocytoma and Paraganglioma
PRAD	Prostate adenocarcinoma
READ	Rectum adenocarcinoma
SARC	Sarcoma
SKCM	Skin Cutaneous Melanoma
STAD	Stomach adenocarcinoma
TGCT	Testicular Germ Cell Tumors
THCA	Thyroid carcinoma
THYM	Thymoma
UCEC	Uterine Corpus Endometrial Carcinoma
UCS	Uterine Carcinosarcoma
UVM	Uveal Melanoma

### Cell culture and reagents

MCF7 and T47D cell lines were obtained from the American Type Culture Collection (ATCC, USA), and they have been verified with short tandem repeat (STR) profiling to be free of mycoplasma. MCF7 cells were cultured in DMEM (11965092, Gibco, USA), while T47D cells were cultured in RPMI-1640 (11875093, Gibco, USA), with 10% fetal bovine serum (FSP500, Excell, China) and 1X penicillin-streptomycin (BL505A, Biosharp, China) added to the culture medium. The cells were incubated at 37 °C in a cell culture incubator with 5% CO_2_ [34]. Palbociclib (PD-0332991) (S1116, Selleck Chemicals, USA) was purchased from Selleck Chemicals, and SC79 (HY-18749, MedChemExpress, USA) was obtained from MedChemExpress. The drugs were dissolved in dimethyl sulfoxide (DMSO, HY-Y0320, MedChemExpress, USA).

### Establishment of palbociclib-resistant cells

MCF7 and T47D cell lines were cultured in media containing palbociclib for six months to generate palbociclib-resistant cell lines, which were named MCF7-PalR and T47D-PalR, respectively. The increasing concentration method was used, starting with a palbociclib concentration of 0.01 μM and maintaining a final concentration of 5 μM [35].

### Transfection and viral infection

TMEM45A-siRNA and negative control siRNA were synthesized by GenePharma Company (Suzhou, China). Cells (1 × 10^5 per well) were seeded in a 6-well plate, and according to the manufacturer’s instructions, siRNA was transfected using Lipofectamine 3000 (L3000001, Invitrogen, USA) for 48 hours. Western blotting and RT-qPCR were used to detect the knockdown efficiency of siRNA. Based on the experimental results, siTMEM45A#1 was selected for subsequent experiments and stable transfection. For stable transfection, siTMEM45A#1 slicing sequence was packed into the lentivirus by GenePharma Company (Suzhou, China). Cells were transfected with the indicated lentivirus and 8 μg/ml polybrene for 48 hours. Puromycin (HY-K1057, MedChemExpress, USA) was added into the medium to select stably transfected cells for at least 14 days. Cells stably transfected with the TMEM45A-targeting lentivirus were named shTMEM45A, and those with the control vector were termed shNC. The sequences of the siRNAs mentioned above are detailed in Table S1.

### Cell counting kit‑8 (CCK‑8) assays, EdU incorporation assay and Colony formation

The cytotoxicity of palbociclib was detected using the CCK-8 assay. Cells (1000 per well) were plated in a 96-well plate and cultured continuously for 5 days with a control reagent or corresponding concentrations of palbociclib. Then, 10 μL of CCK-8 solution (C0038, Beyotime, China) was added to each well and the plate was incubated for an additional 2 hours at 37 °C with 5% CO_2_. The optical density (OD) was then measured at 450 nm using a microplate reader. For the EdU incorporation assay, cells (5×10^5 per well) were seeded in a 6-well plate and treated with either control reagent or 1 μM palbociclib for 24 hours, and then processed according to the instructions of the EdU detection kit (G1640, Servicebio, China). For the colony formation assay, cells (1,000 per well) were seeded in a 6-well plate, treated with either control reagent or 1 μM palbociclib for 14 days, fixed with 4% paraformaldehyde, and stained with crystal violet [10].

### Glucose uptake and lactate production assay

Glucose uptake rate was detected using a flow cytometer with a glucose uptake assay kit (ab235976, abcam, USA). According to the manufacturer’s instructions, the content of lactate was measured using a lactate detection assay kit (E-BC-K044-M, Elabscience, China).

### Immunohistochemistry (IHC)

Paraffin sections were deparaffinized with dimethyl benzene and then washed with 100%, 95%, and 75% ethanol. Antigen retrieval was performed using 0.01 M citrate buffer solution at room temperature, followed by blocking with goat serum for 15 minutes. The sections were incubated with the primary antibody overnight at 4 °C (details of the antibodies are provided in Table S2). The sections were then reacted with biotinylated goat anti-mouse/rabbit IgG polyclonal antibodies and horseradish peroxidase-conjugated streptavidin working solution. The samples were stained with DAB (PH0417, Phygene, China), counterstained with hematoxylin (G1004, Servicebio, China), and then mounted with neutral resin.

Immunohistochemical staining was scored based on the percentage of positive cells and the intensity of staining, with the following scoring criteria: the staining intensity of the cells was graded from 0 to 3, with no positive staining (negative) being 0, pale yellow (weakly positive) being 1, brownish yellow (positive) being 2, and brownish (strongly positive) being 3; the percentage of positive cells in the same field of view was divided into four grades, with a positive rate of ≤25% being 1 point, 26-50% being 2 points, 51-75% being 3 points, and >75% being 4 points. The final immunohistochemical score was obtained by multiplying the two scores [36].

### Western blotting

Cell proteins were extracted by lysing cells on ice with RIPA lysis buffer (P013B, Beyotime, China) containing 1% protease inhibitors and phosphatase inhibitors. After mixing with 5X Loading buffer (P0286, Beyotime, China), the proteins were denatured by continuous boiling in a water bath at 98 °C for 10 minutes. The prepared proteins were then loaded onto SDS-polyacrylamide gels for electrophoresis and transferred onto PVDF membranes. After blocking with 10% BSA for 1 hour, the membranes were incubated with the primary antibody overnight at 4 °C (details of the antibodies are provided in Table S2). Following incubation with the secondary antibodies, anti-mouse/rabbit IgG, at room temperature for 1 hour, the bands were detected using a chemiluminescence detection reagent (BL520A, Biosharp, China). Uncropped blots were shown in Figure Supplementary Original Blots.

### Reverse transcription-quantitative polymerase chain reaction

Total RNA was extracted using the TRIzol reagent (15596026CN, Invitrogen, USA). cDNA was obtained by reverse transcription using the Takara Prime Script RT reagent kit (RR037A, Takara, Japan). Reverse transcription-quantitative polymerase chain reaction (RT-qPCR) was performed using the TB Green Premix Ex Taq II (RR820A, Takara, Japan). The primers used were designed through Primer Bank (https://pga.mgh.harvard.edu/primerbank/). The sequences of all primers are detailed in Table S3.

### Cell cycle analysis and cell apoptosis

Cells (2.5 × 10^5 per well) were seeded in a 6-well plate. After 24 hours of control reagent or 1 μM palbociclib treatment, cell cycle analysis was carried out according to the instructions of the cell cycle detection kit (G1700-50T, Servicebio, China) and the cell apoptosis was determined using an Annexin V apoptosis detection kit (G1510-50T, Servicebio, China). Finally, the samples were analyzed using a flow cytometer (BD FACSVerse, BD Bioscience, USA).

### Transwell migration and invasion assays

The cell migration and invasion capabilities were evaluated using 24-well Boyden chamber model with an 8 μm-pore-size polycarbonate filter (TCS003012, Jetbiofil, China). For the invasion assay, 250 μg/ml of Matrigel (354234, Corning, USA) was applied to the bottom of the chamber. This step was omitted for the migration assay. Then, FBS-free medium was added to the upper chamber, and a medium containing 10% FBS was added to the lower chamber. Cells (2 × 10^4 per well) were seeded in the upper chamber and incubated for 48 hours at 37 °C with 5% CO_2_. Afterward, cells in the upper chamber were wiped off with a cotton swab, and the cells at the bottom of the chamber were fixed with 4% paraformaldehyde and stained with crystal violet for 15 minutes. Images were captured using a microscope.

### Wound healing assays

Cells were seeded in a 6-well plate. When the cell confluence reached 80%, the culture medium was replaced with FBS-free medium and incubated for 24 hours. Subsequently, cells were treated with 5 µg/mL mitomycin C (HY-13316, MedChemExpress, USA) for 2 hours. A scratch was made at the bottom of the well with the tip of a pipette, and images were captured using an inverted microscope. The cells were then incubated for an additional 48 hours in FBS free medium, after which more images were taken using the inverted microscope. The cell migration rate was calculated as follows: % = (wound width at 0 h - wound width at 48 h) / wound width at 0 h × 100%.

### Primary cell culture

HR+ breast cancer specimens were obtained from surgery. Under sterile conditions, excess adipose and connective tissues of the samples were removed. The tissue was digested with Type I and Type III collagenase and hyaluronidase (H3506, Sigma-Aldrich, USA) at 37 °C, stirred in DMEM containing 10% FBS for 3 hours. After standing for 5 minutes, the supernatant was transferred to a new centrifuge tube and centrifuged at 250 *g* for 5 minutes. The primary BRCA cells were then purified using the CD326 (EpCAM) Microbeads kit (130-061-101, Miltenyi Biotec, Germany) according to the kit’s instructions. The primary cells were cultured in DMEM medium containing 20% FBS. The second to fifth generation of primary cells were used in the subsequent experiments [37].

### Isolation of exosomes from primary cells and loading with siRNA

Primary cells were cultured in DMEM containing 20% FBS until they reached 80% confluence. The culture medium was then replaced with exosome-free FBS for 48 hours, after which the supernatant was collected. The supernatant was cleaned through a series of low-speed centrifugations, including 300 × *g* for 10 minutes, 2000 × *g* for 10 minutes, and 10000×g for 30 minutes to remove cells and cell debris; it was then filtered through a 0.22 μm filter. Ultracentrifugation was performed at 4 °C at 100,000 × *g* for 2 hours to pellet the exosomes, which were then washed with pre-chilled PBS.

The siRNA was transfected into exosomes to construct engineered exosome using an electroporation system (Bio-Rad Gene Pluser Xcell, USA). A mixture containing 100 μg of siRNA and 100 μg of purified exosomes was added to 200 μL of electroporation buffer (1.15 mM potassium phosphate pH 7.2, 25 mM potassium chloride, 21% Optiprep), followed by electroporation at 350 V and 150 μF. The mixture was then incubated at 37 °C for 30 minutes to allow the exosome membrane to recover, after which an appropriate amount of PBS was added and the mixture was ultracentrifuged to remove unbound siRNA.

### Characterization and loading Efficiency of siRNA-loaded exosomes

The morphology of the exosomes was examined using a transmission electron microscope (Tecnai G2 Spirit, Thermo Fisher Scientific, USA), and the size and zeta potential of the exosomes, as well as their Polydispersity Index (PDI), were measured using nanoparticle tracking analysis (NTA) and a Zetasizer Nano S90 system (Malvern Instruments, England). The total protein concentration of the exosomes was determined using the BCA protein assay method.

Cyanine-3 (Cy3) labeled siRNA was synthesized by GenePharma (Suzhou, China). To assess the loading efficiency of the engineered exosomes, the fluorescence intensity of Cy3-siRNA at various concentrations was determined using a multimode microplate reader (Bio-Tek Synergy H1, Agilent Technologies, USA). A standard curve correlating siRNA concentration with Cy3 fluorescence intensity was established, along with a concentration calculation formula. The loading efficiency was then computed utilizing the standard curve and the derived formula.

### The biosafety assay of engineered exosomes

To evaluate the stability of the exosomes in blood circulation, different concentrations of exosomes were incubated with whole blood from NOD/SCID mice. PBS solution or ddH_2_O were used as negative or positive controls, respectively. After incubation at 37 °C for 30 minutes, the mixture was centrifuged to observe any hemolytic reaction. At the endpoint of PDX animal experiments, the main organs of the animals were collected for Hematoxylin and Eosin (HE) staining. Blood samples were also collected and tested for ALT (BC1555, Solarbio, China), AST (BC1565, Solarbio, China), BUN (E-BC-K183-M, Elabscience, China), and CREA (E-BC-K188-M, Elabscience, China) according to the instructions provided with the respective assay kits.

### Animal experiments

Female NOD/SCID mice, aged four weeks, were obtained from Gempharmatech Company (Shanghai, China). One week prior to the study, 17β-estradiol pellets (0.72 mg, 60-day release, SE-121, Innovative Research of America, USA) were subcutaneously implanted into the mice [38]. A mixture of 1 × 10^7 MCF7-PalR-shNC or MCF7-PalR-shTMEM45A cells with 10 μL of 250 μg/mL Matrigel (354234, Corning, USA) was then combined with PBS to a final volume of 100 μL and injected into the mammary fat pads of the mice [10]. When the tumor volume approximated 100 mm^3, the mice were treated with the relevant therapeutic agents (*n* = 4 per group). Palbociclib (150 mg/kg, sodium lactate buffer, 50 mM, pH 4.0) was administered 5 days per week by oral gavage, while SC79 was administered at 10 mg/kg/day via intraperitoneal injection [39–42].

Tumor volumes were measured every three days using the formula: Volume = Length × Width^2 × 0.5. After five weeks of continuous treatment, mice were humanely euthanized, the subcutaneous tumors were excised and fixed in 4% paraformaldehyde prior to being embedded in paraffin for immunohistochemistry analysis.

### PDX model

A patient-derived xenograft (PDX) model was established to assess the therapeutic efficacy of Exo-siTMEM45A. Clinical specimens were obtained from the Third Affiliated Hospital of Sun Yat-sen University. Fresh untreated ER+, PR+, HER2- primary invasive breast cancer tissue specimens were collected, the information and clinical pathological details of the patient who constructed the PDX model is detailed in Table S4. The specimens were cut into 3×3×3 mm fragments, and 3 fragments were subcutaneously implanted into each NOD/SCID mouse, named PDX-G1. When the volume of the PDX-G1 generation mice reached approximately 150–200 mm^3, the mice were randomly divided into the parent group (PDX-pa) and the palbociclib resistant group (PDX-PalR). The PDX-PalR group was treated with palbociclib (palbociclib 150 mg/kg, sodium lactate buffer, 50 mmol/L, pH 4.0, administered 5 days per week by oral gavage). When the tumor volume of the control group reached approximately 1000 mm^3, the tumor tissue was excised and passaged [23]. The volume of the fourth-generation PDX tumor (PDX-G4) was about 125–150 mm^3, the PDX-pa and PDX-PalR were randomly divided into six groups each (*n* = 3 each), and then the mice were treated for five weeks with the relevant therapeutic agents (palbociclib 150 mg/kg or 50 mg/kg, sodium lactate buffer, 50 mmol/L, pH 4.0, administered 5 days per week by oral gavage; exosomes or exo-siTMEM45A were injected via the tail vein, 20 μg/dose, every three days). One week before the tumor tissue was transplanted into the mice, 17β-estradiol pellets (0.72 mg, 60-day release, SE-121, Innovative Research of America, USA) were implanted subcutaneously into the mice. Tumor volumes were measured every three days. All the above animal experiments were reviewed and approved by the Ethics Committee of Guangdong Provincial People’s Hospital, with the approval number: KY2024-336-01.

### IVIS Lumina imaging

To evaluate the targeting of engineered exosomes in vivo, when the tumor volume of PDX-G4 control group mice was approximately 500 mm^3, free siNC or engineered exosomes were injected, and in vivo imaging was performed using the IVIS Lumina system (Perkin Elmer, USA) at 4-, 24-, and 48-hours post-injection.

### Statistical analysis

All experiments were analyzed using GraphPad Prism v.8.0 software for statistical analysis. Experimental data are presented as the mean ± standard deviation (SD). The Student’s T-test was used for analyzing two groups, while one-way analysis of variance (ANOVA) was employed for three or more groups. Overall survival (OS) was compared using the Kaplan-Meier method and the log-rank test. Correlation analysis between different factors was performed using Spearman’s correlation analysis. A *P*-value of less than 0.05 was considered to indicate statistical significance. Compared to the control group, “ns” indicates no statistical significance; * indicates *P* < 0.05; ** indicates *P* < 0.01; *** indicates *P* < 0.001.

## Supplementary information

Supporting Information

Supplementary Original Blots

## Data Availability

All data necessary to assess the conclusions of the work are included in the paper and/or the Supplementary Materials. Additional data relevant to this article are available from the corresponding author upon request.
