# Supplementary material for: TMEM45A enhances palbociclib resistance and cellular glycolysis by activating AKT/mTOR signaling pathway in HR+ breast cancer
Source: Cell Death Discov. 2025 Feb 5;11:47. doi: 10.1038/s41420-025-02336-9 (PMC11799145; doi:10.1038/s41420-025-02336-9)
Supplement: Supplementary file 1 — Supporting Information [file 41420_2025_2336_MOESM1_ESM.docx]

**Supporting Information**

**
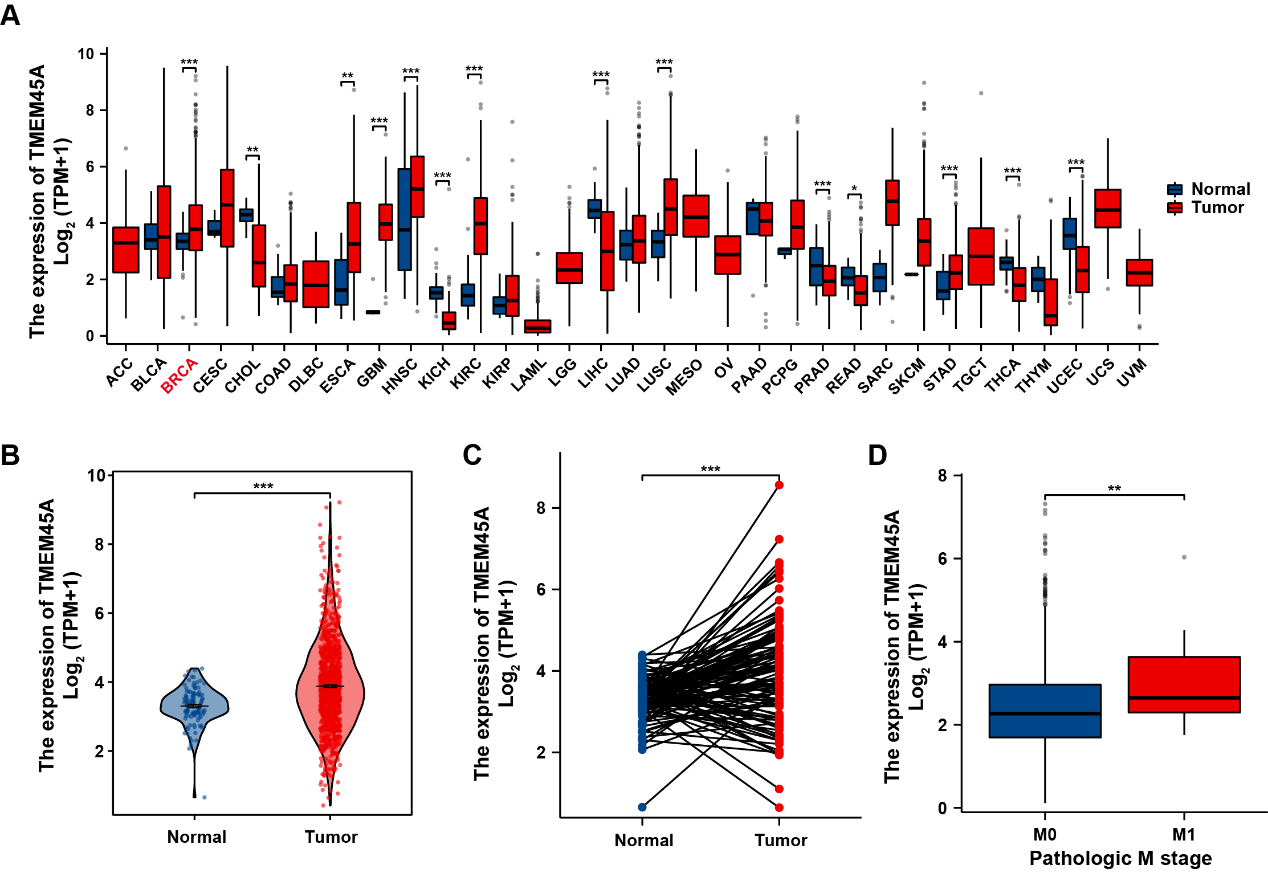
**

**Figure S1. Pan-cancer expression profiling analysis of TMEM45A.**

A. Comparison of TMEM45A mRNA expression in tumor tissue and adjacent normal tissue in the TCGA database. The abbreviations and full names of the cancers mentioned are listed in Table 1.

B. TMEM45A expression in BRCA tissues as compared to the noncancerous breast tissues from the TCGA.

C. Difference in expression of TMEM45A between BRCA and matched normal tissues in TCGA data sets.

D. TMEM45A expression levels and the pathological M stages.

*P < 0.05, **P < 0.01, ***P < 0.001.


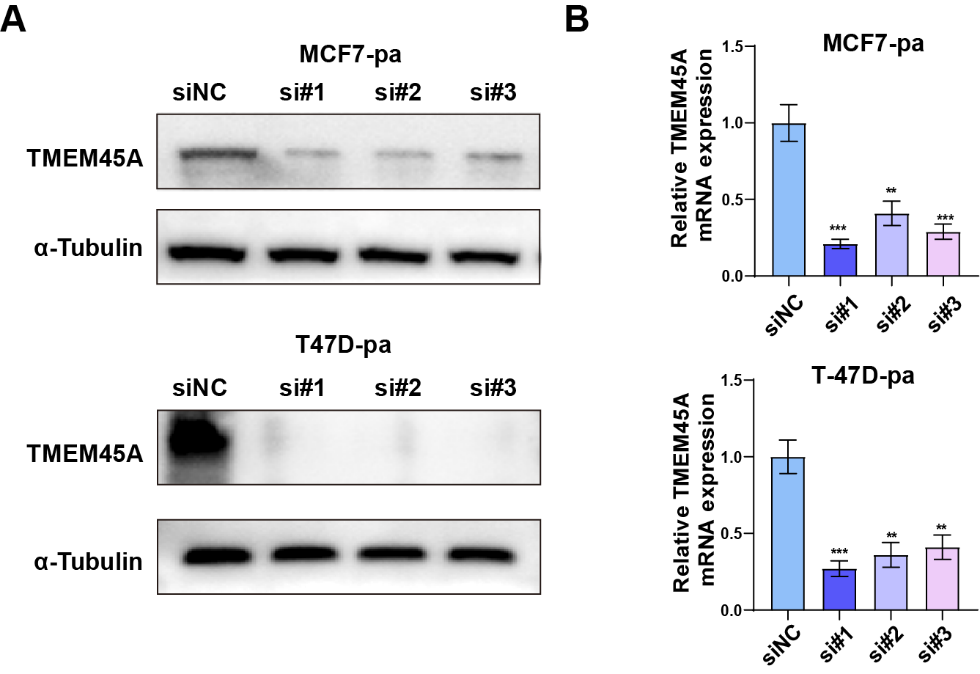


**Figure S2. TMEM45A knocking out efficiency by in BRCA cells.**

A and B. (A) Western blotting and (B) RT-PCR show the knockdown efficiency of TMEM45A.

**P < 0.01, ***P < 0.001.


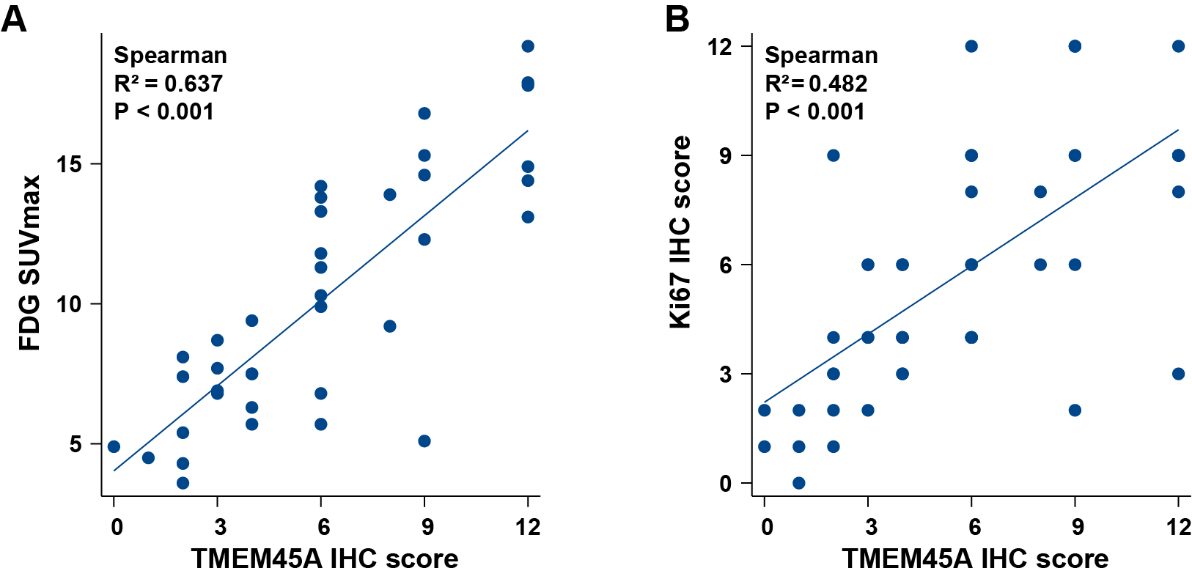


**Figure S3. TMEM45A expression was positively correlated with 18F-FDG accumulation and Ki67 expression.**

A and B. Spearman correlation of TMEM45A with (A) 18F-FDG accumulation and (B) the expression levels of Ki67.


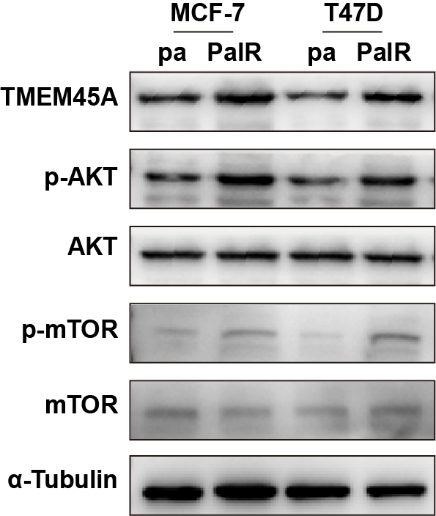


**Figure S4. AKT/mTOR pathway was activated in palbociclib-resistant BRCA cells.**

Western blot showing the AKT/mTOR pathway in MCF7-PalR and T47D-PalR cells and their parental cells.


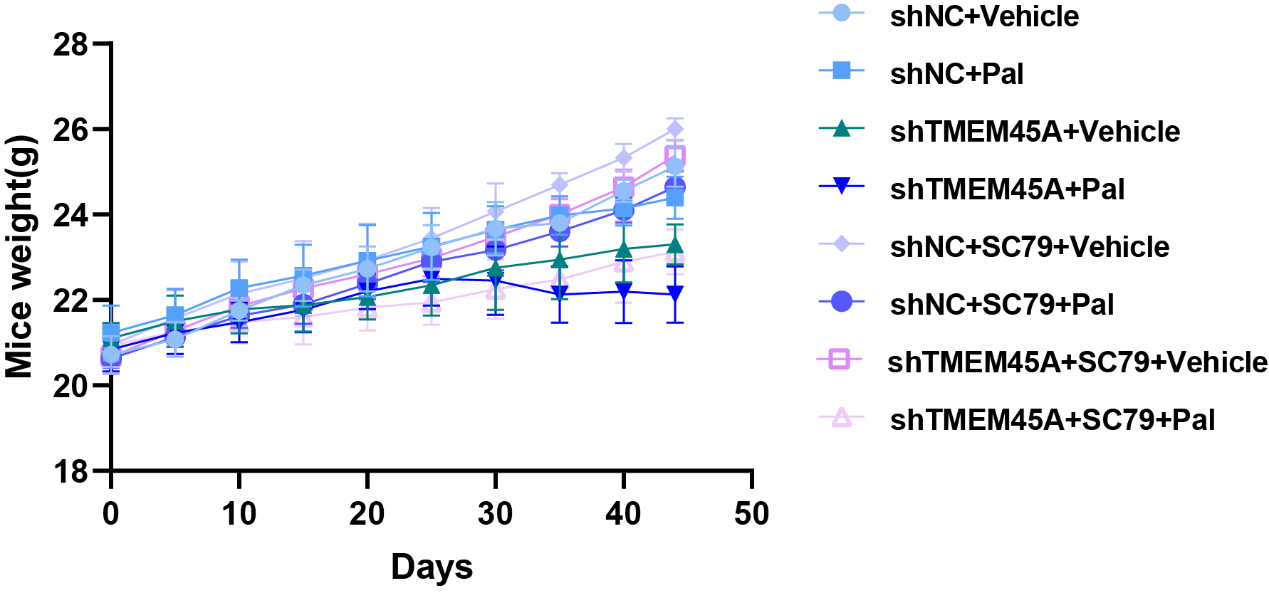


**Figure S5. The weights of mice in each group with different treatments.**

**
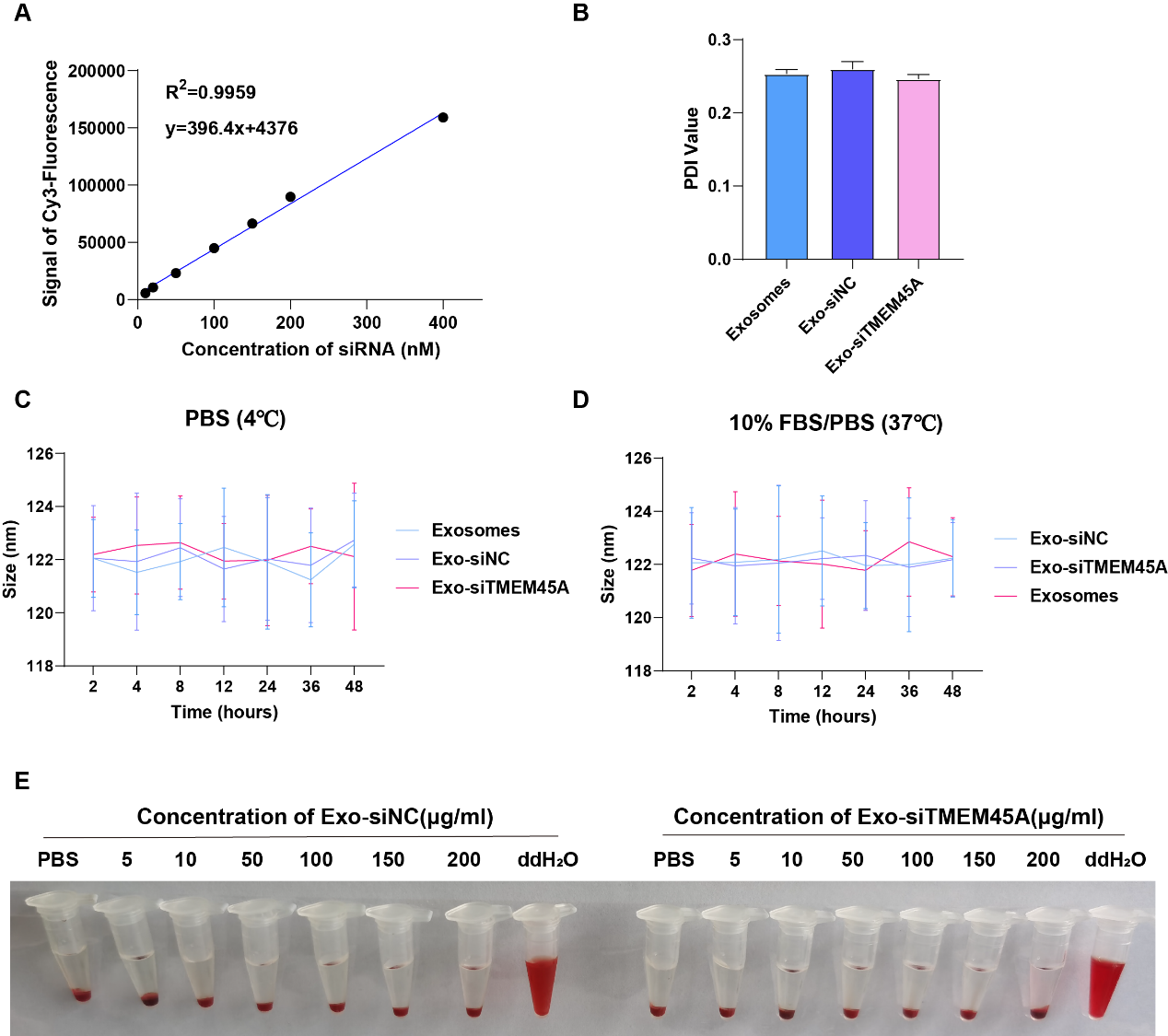
**

**Figure S6. The** **standard curve, PDI, stability and biocompatibility of engineered exosomes.**

A. The standard curve correlating siRNA concentration with the intensity of Cy3 fluorescence signals.

B. The PDI value of exosomes, Exo-siNC and Exo-siTMEM45A.

C and D. The stability test of exosomes in (C) PBS (4℃) and (D) 10% FBS (37℃).

E. Engineered exosomes were incubated with whole blood of NOD SCID mice at different concentrations and no hemolytic reaction was observed.


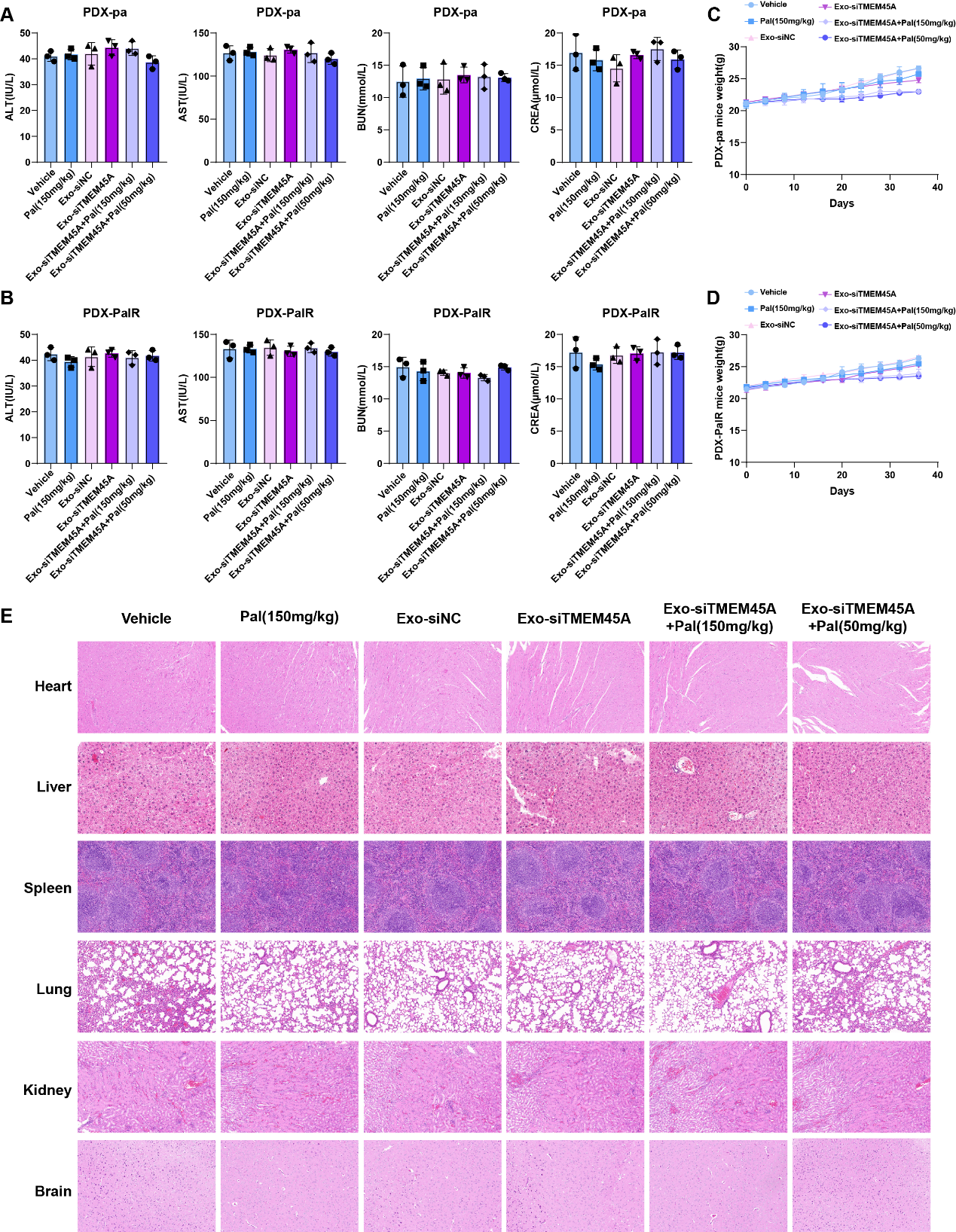


**Figure S7. Biosafety evaluation of the engineered exosomes in a PDX model.**

A and B. Assessments of BUN, CREA, AST, and ALT in the serum demonstrated no significant alterations among groups.

C and D. The weights of mice in each group with different treatments of PDX.

E. HE staining analysis showed no evident toxicity in organs, and all the PDX mice remained alive by the end point of the in vivo experiments.

**Table S1 |Sequence of siRNAs used in the research.**

| siRNA | Guide (5′→3′) | | Passenger (5′→3′) |
| --- | --- | --- | --- |
| siTMEM45A#1 | | UUUAACCAAAUUGGAAAUGTT | CAUUUCCAAUUUGGUUAAATT |
| siTMEM45A#2 | | AAUGGUUACUGCAUAAUGCTT | GCAUUAUGCAGUAACCAUUTT |
| siTMEM45A#3 | | AGUCAUAUAACAUCAGAUGTT | CAUCUGAUGUUAUAUGACUTT |
| Negative Control(siNC) | | UUCUUCGAACGUGUCACGUTT | ACGUGACACGUUCGGAGAATT |

**Table S2 |Antibodies and its dilutions**

| Antigen | Host species | Dilution | Manufacturer and Cat. No. | Application |
| --- | --- | --- | --- | --- |
| TMEM45A | Rabbit | 1:1000 | Abcam, ab166899 | WB |
| TMEM45A | Rabbit | 1:50 | Sangon Biotech, D263782 | IHC |
| Ki67 | Rabbit | 1:500 | Servicebio, GB111141-100 | IHC |
| N-Cadhein | Mouse | 1:10000 | Proteintech,66823-1-lg | WB |
| E-Cadhein | Rabbit | 1:1000 | Cell SignalingTechnology,3195S | WB |
| Vimentin | Rabbit | 1:1000 | Cell Signaling Technology,5741S | WB |
| Snail | Rabbit | 1:1000 | Cell Signaling Technology,3879S | WB |
| GPI | Rabbit | 1:1000 | Proteintech,15171-1-AP | WB |
| PGAM1 | Rabbit | 1:2000 | Proteintech,13126-1-AP | WB |
| PKM2 | Rabbit | 1:1000 | Proteintech,1582-1-AP | WB |
| p-AKT | Rabbit | 1:2000 | Proteintech,66444-1-AP | WB |
| p-AKT | Rabbit | 1:100 | Proteintech,66444-1-AP | IHC |
| AKT | Rabbit | 1:5000 | Proteintech,60203-2-AP | WB |
| p-mTOR | Mouse | 1:2000 | Proteintech,67778-1-lg | WB |
| p-mTOR | Mouse | 1:500 | Proteintech,67778-1-lg | IHC |
| mTOR | Mouse | 1:5000 | Proteintech,66888-1-lg | WB |
| α-Tubulin | Rabbit | 1:1000 | Cell Signaling Technology,2144S | WB |
| Anti-rabbit IgG | Mouse | 1:5000 | Sangon Biotech, D110065 |  |
| Anti-mouse IgG | Rabbit | 1:5000 | Sangon Biotech, D110098 |  |

**Table S3 |Sequence of qRT-PCR Primers used in the research.**

| Gene | Species | Forward(5'-3') | Reverse(5'-3') |
| --- | --- | --- | --- |
| TMEM45A | Human | TTATGCAGTAACCATTGTCATCGTT | TGATTCTTGTTCTCGTTCAGCATT |
| GLUT1 | Human | CATCCCATGGTTCATCGTGGCTGAACT | GAAGTAGGTGAAGATGAAGAACAGAAC |
| HK2 | Human | GCCATCCTGCAACACTTAGGGCTTGAG | GTGAGGATGTAGCTTGTAGAGGGTCCC |
| GPI | Human | TATTGTGTTCACCAAGCTCACACC | TGGTAGAAGCGTCGTGAGAGGTC |
| PFKL | Human | GGAGAAGCTGCGCGAGGTTTAC | ATTGTGCCAGCATCTTCAGCATGAG |
| ALDOA | Human | AGGCCATGCTTGCACTCAGAAGT | AGGGCCCAGGGCTTCAGCAGG |
| GAPDH | Human | TTCCGTGTCCCCACTGCCAACGT | CAAAGGTGGAGGAGTGGGTGTCGC |
| PGK1 | Human | ATGTCGCTTTCTAACAAGCTGA | GCGGAGGTTCTCCAGCA |
| PGAM1 | Human | GGAAACGTGTACTGATTGCAGCCC | TTCCATGGCTTTGCGCACCGTCT |
| ENO1 | Human | GACTTGGCTGGCAACTCTG | GGTCATCGGGAGACTTGAA |
| PKM2 | Human | GCCCGTGAGGCAGAGGCTGC | TGGTGAGGACGATTATGGCCC |
| LDHA | Human | ATGGCAACTCTAAAGGATCA | GCAACTTGCAGTTCGGGC |
| α-Tubulin | Human | CCAAGCTGGAGTTCTCTA | CAATCAGAGTGCTCCAGG |

**Table S4 |Clinical characteristics on patients whose tumors were collected for establishing PDX model.**

| Patient | Age | Gender | Pathological Diagnosis | Subtype | Clinical TNM stage |
| --- | --- | --- | --- | --- | --- |
| Patient#1 | 58 | Female | Infiltrating carcinoma | ER(+), PR(+),HER2(-) | T2N1M0 |
