# Supplementary figures and images for: TMEM45A enhances palbociclib resistance and cellular glycolysis by activating AKT/mTOR signaling pathway in HR+ breast cancer
Source: Cell Death Discov. 2025 Feb 5;11:47. doi: 10.1038/s41420-025-02336-9 (PMC11799145; doi:10.1038/s41420-025-02336-9)

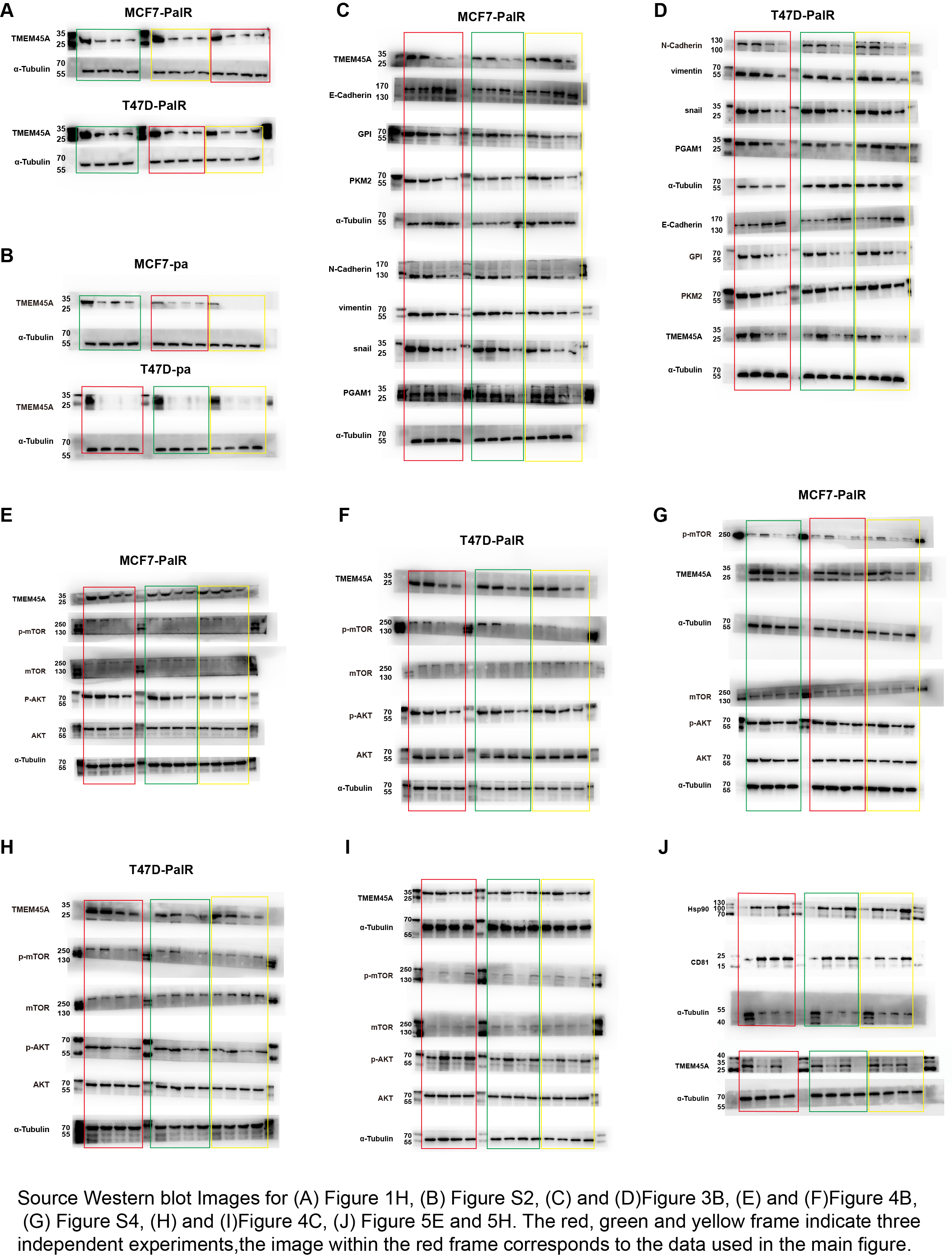

Supplement: Supplementary file 2 — Supplementary Original Blots [file 41420_2025_2336_MOESM2_ESM.tif]
